# Kinetically Controlled Synthesis of Non-Noble Metal Based High-Entropy Alloy Nanoparticles via Supersonic-Nozzle-Assisted Thermal Plasma Jet

**DOI:** 10.1021/acsnano.6c02465

**Published:** 2026-07-08

**Authors:** Ziqi Tang, Martin Couillard, Jian Chen, Homin Shin, Olga Naboka, James Tordiff, Jae-Young Cho, Thomas Lacelle, Dean Ruth, Mark Plunkett, Michel Nganbe, Keun Su Kim

**Affiliations:** 1 Quantum and Nanotechnologies Research Centre, National Research Council Canada, Ottawa, ON K1A 0R6, Canada; b Quantum and Nanotechnologies Research Centre, National Research Council Canada, Edmonton, AB T6G 2M9, Canada; 2 Department of Mechanical Engineering, University of Ottawa, Ottawa, ON K1N 6N5, Canada; 3 Department of Mechanical and Industrial Engineering, University of Toronto, Toronto, ON M5S 3G8, Canada; 4 Clean Energy Innovation Research Centre, National Research Council Canada, Ottawa, ON K1A 0R6, Canada; 5 Construction Research Centre, National Research Council Canada, Ottawa, ON K1A 0R6, Canada; 6 Department of Mechanical Engineering, University of Alberta, Edmonton, AB T6G 1H9, Canada

**Keywords:** high-entropy alloy nanoparticles, thermal plasma, supersonic nozzle, ultrafast quenching, kinetically controlled synthesis

## Abstract

The scalable synthesis of high-entropy alloy nanoparticles (HEA NPs) with atomic-level elemental homogeneity still remains a significant challenge, particularly for systems with high binary mixing enthalpies. Here, we demonstrate continuous, high-volume (∼5 g/h) synthesis of compositionally uniform, single-phase HEA NPs with average sizes around 30 nm using a supersonic-nozzle-assisted thermal plasma jet. The nozzle provides significantly enhanced jet velocities up to the supersonic regime (*M*_a_ > 1) with highly directional flow to achieve uniform, ultrafast cooling rates of up to 10^8^ K/s. This ultrafast quenching effectively limits atomic diffusion, thereby suppressing elemental segregation even in systems with high binary mixing enthalpies (e.g., Cu–Cr: 12 kJ/mol). The extreme cooling rates also impact the growth of HEA NPs, resulting in crystallite size reduction and lattice parameter contraction. Finally microstructural analysis reveals that the supersonic nozzle leads to a distinct nanostructure transition from long-range, uniform to nonuniform nanotwins, providing a mechanism for tuning the structure of HEA NPs at nanoscales.

## Graphical abstract

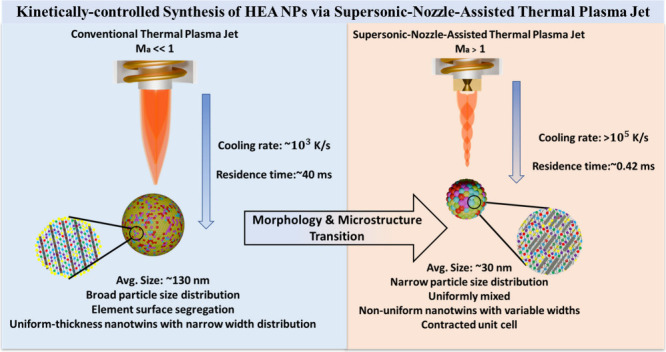

## Introduction

As technologies become more complex and highly integrated, material innovation with multifunctionality is highly desirable for cost efficiency and sustainability. In the past decade, chemical/compositional complexity in nanoalloys has increased drastically to integrate different functions into a single material system and achieve unconventional performance.^1^ Prominent examples are high-entropy alloy nanoparticles (HEA NPs) composed of five or more principal elements in near-equimolar ratios. They can exhibit a unique set of properties distinct from existing conventional alloys, thanks to the combination of their compositional variety and confinement effects at nanoscales.^2–4^ These new engineered materials provide unprecedented opportunities for the development of new advanced technologies in various fields, including structural alloys, catalysis, energy conversion/storage, sensors, and biomedical applications.^5–7^

Recent years have witnessed notable progress in the synthesis of HEA NPs via various synthesis routes including thermal shock, hydrothermal, pulsed/scanning laser ablation, aerosols, continuous flow reactor, rapid joule heating, plasmas and so on.^8–14^ The current research primarily focuses on the precise control over the HEA NP composition, phase, size and morphology with targeted properties to harness their full potential in real-world applications.^15–18^ Achieving elemental homogeneity of HEA NPs remains one of the critical challenges, particularly for compositions involving elements with high positive binary mixing enthalpies (e.g., Cu–Cr, Cu–Fe, Zn–Fe). For example, the formation of Cu–Ni enriched phases has been observed in HEA NPs produced by laser ablation, and Cu surface segregation is a persistent problem in the plasma arc discharge synthesis of CrFeCoNiCu systems.^14,19^ Atomic-level numerical studies further confirm that Cu surface segregation is an energetically favorable process in HEAs at the molten stage.^20^

Thermal shock or wet-chemical-based methods are currently among the most promising approaches for HEA NP synthesis. They typically utilize a mixture of metal salts as precursors and enable atomic-level homogeneous mixing of the constituent elements prior to HEA NP nucleation and growth. In the thermal shock method, the rapid heating–cooling cycle creates conditions far away from thermal equilibrium and thus allows co-reduction of metal precursors and subsequent nucleation and growth of HEA NPs without elemental segregation. While this method successfully demonstrated a diverse library of HEA NPs with precise control over nanoparticle sizes and compositions, the process is often operated in the batch mode limited at the small scales (<1 g/h).^4^ The wet-chemical-based synthesis is a well-established method and highly suited for the large-scale synthesis of HEA NPs.^21^ However, nonsynchronized or sequential reduction of metal precursors may arise due to reduction potential gaps, leading to inhomogeneous elemental distributions in HEA NPs.^22^

Very recently, we reported a thermal plasma process for the HEA NP synthesis to address the shortcomings of the above approaches.^23^ This one-step process allows the continuous synthesis of HEA NPs at a high production rate from a mixture of pure elemental metal powders using an inductively coupled plasma jet (ICPJ). This new process avoids the reduction step by using pure elemental metals. However, it also introduces new challenges arising from nucleation temperature gaps among the constituent metal elements and insufficient cooling rates, which may lead to compositional variations in HEA NPs and, more critically, elemental segregation (e.g., Cu segregation in CrFeCoNiCu HEA NPs). The process also suffers from difficulties in limiting the particle size below 50 nm and achieving narrow particle size distributions due to particle coagulation prior to their consolidation and a strong radial gradient in the thermofluidic field, respectively.

In this study, we employ a supersonic-nozzle-assisted inductively coupled plasma jet (ICPJ) system that provides significantly enhanced jet velocities with highly directional flow to achieve uniform, ultrafast cooling rates of 10^6^–10^8^ K/s. This rapid cooling is critical to prevent element segregation during the in-flight alloying process, thereby promoting the formation of compositionally uniform, single-phase HEA NPs even in thermodynamically unfavorable systems. Thanks to the short growth time of NPs with the highly directional flow, the supersonic nozzle also facilitates the formation of smaller size NPs with narrow size distributions. We report the synthesis of four distinct HEA NP compositions (CrFeCoNiCu, MnFeCoNiCu, MnFeCoNiZn, and MnCoNiCuZn) with average particle sizes around 30 nm, achieved at a high production rate of ∼5 g/h. Despite the high positive binary mixing enthalpies (e.g., Cu–Cr, 12 kJ/mol; Cu–Fe, 13 kJ/mol; Cu–Co, 6 kJ/mol),^24^ all five elements in each HEA system are homogeneously mixed at the atomic scale with good crystallinity. We systematically investigate the effect of the supersonic nozzle on particle morphology, microstructure, and composition, demonstrating that this approach substantially expands the composition space for the continuous synthesis of HEA NPs, including HEA systems that are often thermodynamically prone to segregation.

## Results and Discussion

### Synthesis of HEA NPs with a Converging–Diverging (CD) Supersonic Nozzle

Figure 1 shows the schematics of the ICPJ process developed and compares the HEA NP synthesis with and without a nozzle. In this plasma process, the solid metal precursors are rapidly heated and vaporized at temperatures exceeding 5000 K, forming a well-mixed, multicomponent metal vapor. As the plasma jet expands at the reactor entrance, the vapor cools to a supersaturated state, leading to homogeneous nucleation of NPs, typically initiated by the metal element with the highest nucleation temperature. The nucleated NPs continue to grow in the liquid phase through cocondensation of metal monomers. During this growth stage, atomic diffusion within the particles promotes the alloying process. Finally, rapid quenching solidifies the NPs, preserving their homogeneous, well-mixed composition.

**Figure 1:**
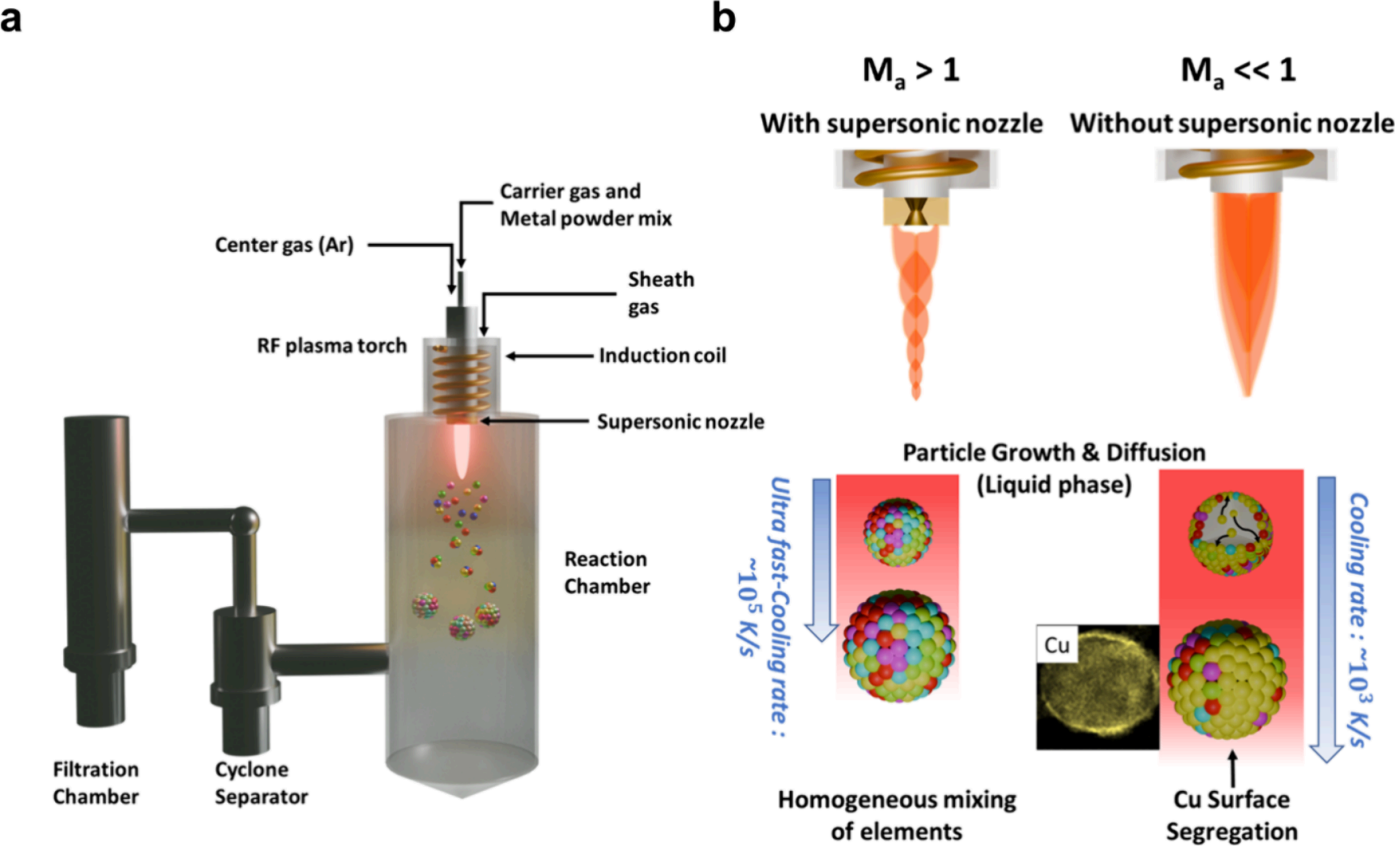
(a) Schematic of the supersonic-nozzle-assisted inductively coupled plasma jet (ICPJ) process developed for the continuous synthesis of HEA NPs. (b) Comparison of the ICPJ process with and without a supersonic nozzle where *M*_a_ is the Mach number. In our previous work,^23^ Cu segregation was observed in CrFeCoNiCu HEA NPs when the nozzle was not employed. Inset: Cu elemental map.

In our previous work, the thermal plasma generated expands at the entrance of the reactor without other special means and mixes with the cold ambient gas, leading to the natural cooling of the reaction stream. While such uncontrolled cooling successfully demonstrated the synthesis of various HEA NPs with a high production rate, the process favors the production of relatively large NPs with a broad size distribution. We also found Cu segregation at the surfaces of particles when Cr is included (inset of Figure 1). This is attributed to the limited solubility between Cr and Cu at equilibrium and in line with the trends reported in other HEA systems (Table S1).^14,19,25^ The Cu segregation seems to be prohibited as the particle size decreases, indicating that smaller particle sizes with narrow size distributions would be favorable to achieve a good compositional uniformity in this plasma process.^23^

In the ICPJ process, the growth of smaller particles is feasible by rapid cooling of the vapor precursors. This allows maintaining a highly supersaturated state prior to the onset of nucleation, thereby enabling the formation of more abundant smaller nuclei. On the other hand, a narrow size distribution is achievable via uniform quenching where the nuclei formed in the reactor travel within a similar thermal history. The vapor precursors could be further quenched by mixing with the cold gas injected via porous reactor walls or auxiliary gas ports. However, this is an inherently inhomogeneous cooling process accompanying complex flow and thus often results in the production of NPs with broad size distributions. Alternatively, a controlled expansion of the plasma jet using a nozzle not only provides a high quenching rate but also generates highly directional flow approaching the quasi-one-dimensional flow.^26^ This allows achieving reasonably uniform temperature, velocity, and concentration profiles along the radial direction except for the thin boundary layer of the plasma jet.

In this work, a converging–diverging (CD) nozzle was designed and fabricated based on the quasi-one-dimensional compressible flow theory so that the plasma jet can expand up to the supersonic regime through expansion (see the nozzle design section in Supporting Information and Figure S1). The geometry of the CD nozzle is shown in Figure S2. During synthesis experiments, the plasma undergoes supersonic expansion into a reaction chamber, with a static pressure drop from ∼66.7 kPa to 22.7 kPa. This expansion results in a significant increase in gas velocity while reducing temperature and residence time in the particle growth zone, so that the multicomponent vapor undergoes uniform and rapid cooling with a high quenching rate up to 10^8^ K/s. This condition not only allows high degrees of vapor saturation leading to the production of smaller particles but also provides an unusual synthesis environment far away from chemical and thermal equilibrium (e.g., conucleation from the multicomponent vapor, homogeneous mixing of immiscible elements). In our previous work, both helium and hydrogen were utilized as the plasma gas.^23^ In the present work, we consider only hydrogen as the plasma gas due to the high cost of helium. More details on the synthesis condition can be found in Experimental Section.

### Morphology, Structure, and Composition Characterization

Figure 2a shows a SEM image of the as-produced CrFeCoNiCu HEA NPs with the nozzle (Figure S3 shows more SEM images at different magnifications and also compares them with SEM images of the HEA NPs produced without the nozzle). We focus on this specific HEA system because it exhibits Cu segregation in our previous work.^23^ The HEA NPs are spherical in shape and exhibit no considerable physical aggregation/agglomeration (also seen in TEM image in Figure 2b). The average particle size is estimated around 31.1 nm based on SEM analysis (Figure 2c), which is much smaller than that of the HEA NPs synthesized without the nozzle (128.5 nm, Figure S4). The particle size refinement can be rationalized by the ultrafast cooling rate and shorter residence time in the growth zone achieved by the nozzle. In addition, the highly directional flow created by the nozzle leads to uniform cooling of precursors and thus minimizes the broadening of the particle size distribution. Similar trends in the morphology and size distribution are observed for the other three HEA NPs (MnFeCoNiCu, MnFeCoNiZn, and MnCoNiCuZn HEA NPs) synthesized with the nozzle (Figure S5 for SEM images and Figure S6 for particle size distributions).

**Figure 2:**
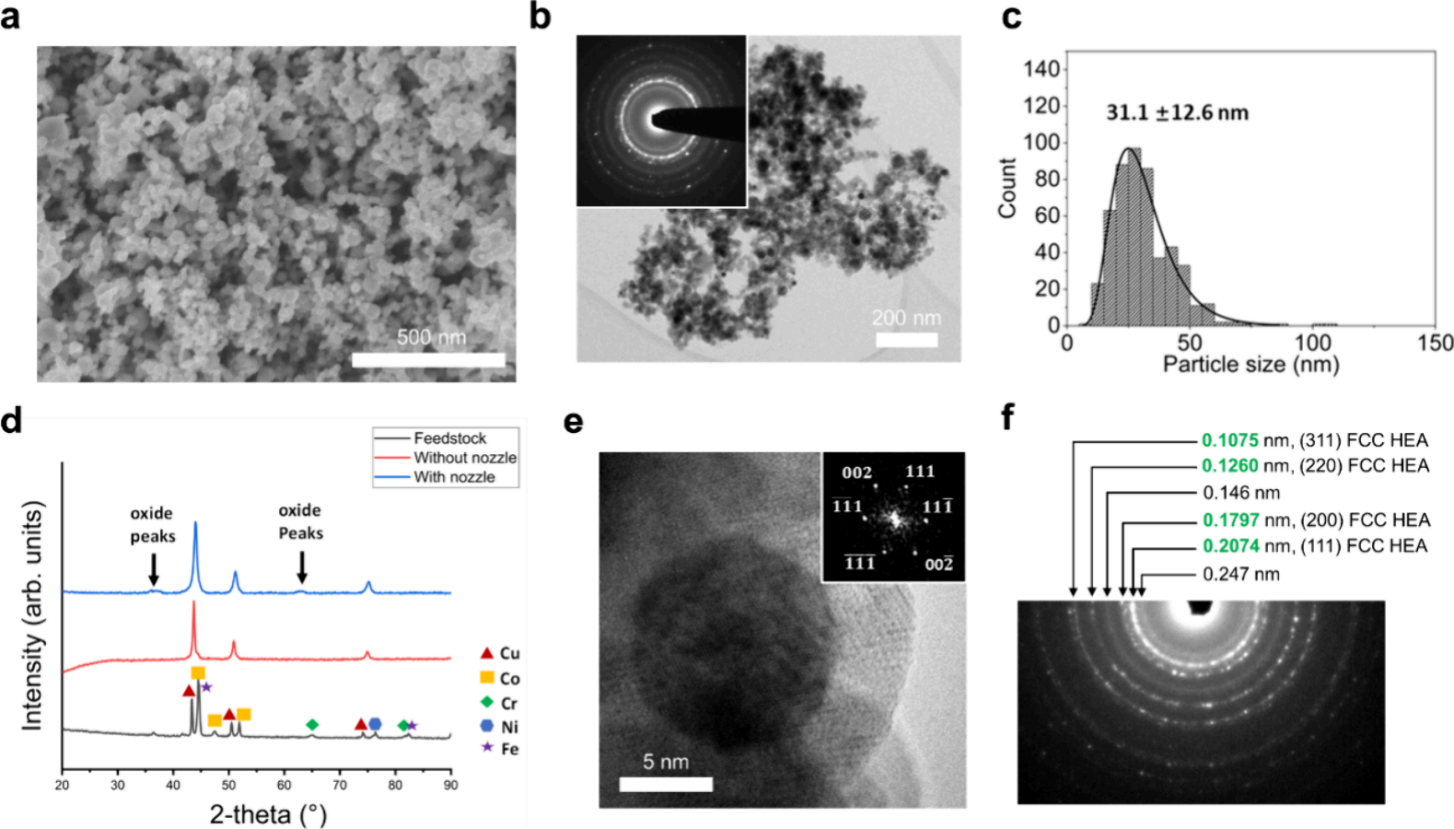
Morphological and structural characterization of CrFeCoNiCu HEA NPs produced with the supersonic nozzle. (a) SEM image. Scale bar, 500 nm. (b) TEM image and SAED pattern at low magnification. Scale bar, 200 nm. (c) Particle size distribution. (d) XRD pattern (blue line). Red and black lines are XRD patterns of the sample produced without the nozzle and its feedstock mixture, respectively. (e) HR-TEM image of a HEA NP and its FFT diffractogram. Scale bar, 5 nm. (f) SAED image and identification of ring patterns. The two *d* spacings (i.e., 0.247 and 0.146 nm) can be assigned to the (311) and (440) plane of CrFeCoNiCu oxide, respectively, according to the published results.^32^

XRD patterns of CrFeCoNiCu HEA NPs produced with and without nozzle are shown in Figure 2d along with that of the feedstock. Figure S7 provides XRD patterns of other samples produced with different elemental compositions. All HEA samples exhibit a highly crystalline, single face-centered cubic (FCC) crystal structure with distinct diffraction peaks at 44.0° (111), 51.2° (200), and 75.1° (220), without detectable phase separation. A minor diffraction peak around 36° is commonly observed for all four samples synthesized with the nozzle (indicated with arrows in the corresponding XRD patterns). This peak matches well with the strongest peak from the (311) plane of (CrMnFeCoNi)_3_O_4_ reported in literature^27^ and may indicate the presence of CoCr_2_O_4_ or Cr_2_O_3_. Since the NPs were synthesized in a reducing environment using an Ar–H_2_ plasma gas, the formation of the oxide phases is most likely due to postsynthesis surface oxidation upon exposure to air. A recent study on the oxidation of HEA NPs reported that the oxide layer grows with a continuously decreasing oxidation rate, suggesting that this postsynthesis oxidation is self-limiting.^28^ This peak is not visible in the sample produced without the nozzle. Since a higher surface to volume ratio usually leads to the formation of more oxide phases at the surface, this observation indicates the smaller size of the HEA NPs synthesized with the nozzle.

Since the constituent elements and their ratios are different across the HEA NP samples synthesized with the nozzle, the positions of the diffraction peaks are slightly different. The lattice constants estimated from the corresponding XRD patterns are 3.578 Å (CrFeCoNiCu), 3.605Å (MnFeCoNiCu), 3.565Å (MnFeCoNiZn), and 3.599Å (MnCoNiCuZn), respectively (Table S2). For the CrFeCoNiCu HEA NP sample, the lattice constant is in close agreement with the value of 3.588–3.602 Å reported in literature where NPs were synthesized using a pulsed laser ablation method.^29^ The lattice constants of HEA NP samples synthesized without the nozzle are comparatively summarized in Table S3. It can be seen that with the nozzle, the lattice constants are slightly smaller with differences within 0.01 Å. This lattice contraction is attributable to the increased surface stress in samples synthesized with the nozzle.^30^ More rapid thermal quenching may result in significant particle size refinement (∼30 nm), which can induce greater internal compressive stresses via increased surface tension.^31^ Lastly, the interplanar spacings of each plane for all the samples are summarized in Table S4. A consistent reduction in *d* spacing is observed for the samples synthesized with the nozzle, confirming the lattice contraction induced by rapid solidification.

The high-resolution TEM (HR-TEM) image of a CrFeCoNiCu NP (Figure 2e) with its fast Fourier transform (FFT) diffractogram suggests a single-phase, solid-solution structure. The particle also exhibits core–shell structure. The shell can be identified as oxide layers, based on the oxide peaks in the XRD patterns. The selected area electron diffraction (SAED) pattern (Figure 2f) of the corresponding sample also exhibits diffraction rings pattern that can be indexed to the (111), (200), (220), and (311) planes of the FCC structure. Two faint rings can be observed in the ring pattern with *d* spacings of 2.47 Å and 1.46 Å, respectively. They can be assigned to (311) and (440) planes of the oxide of CrFeCoNiCu, respectively.^32^

Figure 3 presents the elemental maps acquired using electron energy loss spectroscopy (EELS) under STEM mode for individual and multiple NPs synthesized with the nozzle (see also Figure S8 for SEM-EDX elemental maps of all the samples with various particle sizes). Regardless of their compositions, most of the particles show homogeneous mixing of their five elements without significant elemental or phase segregation (Figure 3a–-d). However, some elemental maps reveal a nonuniform distribution of certain elements, notably a “halo-like” enrichment of Fe or Mn at the particle surface (indicated by white arrows in Figure 3a,b). This may be attributed to postsynthesis surface oxidation, which is evident from O spectrum images (Figure 3a,b for a single NP; Figure S9 for multiple NPs). Elements with a high affinity toward oxygen, such as Mn and Fe, are thermodynamically driven to migrate from the homogeneous metallic core to the surface via solid-state diffusion, in order to participate in the oxidation.^33,34^ The thickness of the oxide layer and its volume content are estimated to be around 5 nm and 40%, respectively, which is much higher than those of the samples produced without the nozzle (20%), indicating their smaller particle sizes and larger surface-to-volume ratio that favors oxidation. The presence of such surface oxides may influence the applicability of the HEA NPs. For applications requiring a purely metallic surface, the oxide layer may represent a drawback, as it can hinder direct access to the metal’s active sites. The oxide layer can be removed through post-treatment in a strong reducing environment, such as nonthermal hydrogen plasma.^35,36^ Conversely, the oxide shell can act as a protective layer against corrosion, and thus the metal–oxide core–shell structure can exhibit unique catalytic performance improving both activity and durability.^12,37^ Small Mn rich particles are also observed (indicated by yellow arrows in Figure 3b,f). Considering Mn’s high vapor pressure, it is assumed that Mn vaporized stays longer in the vapor phase and deposits as discrete manganese on the side of the particle,^26^ although overall elemental distributions in the HEA NPs remain largely uniform.

**Figure 3:**
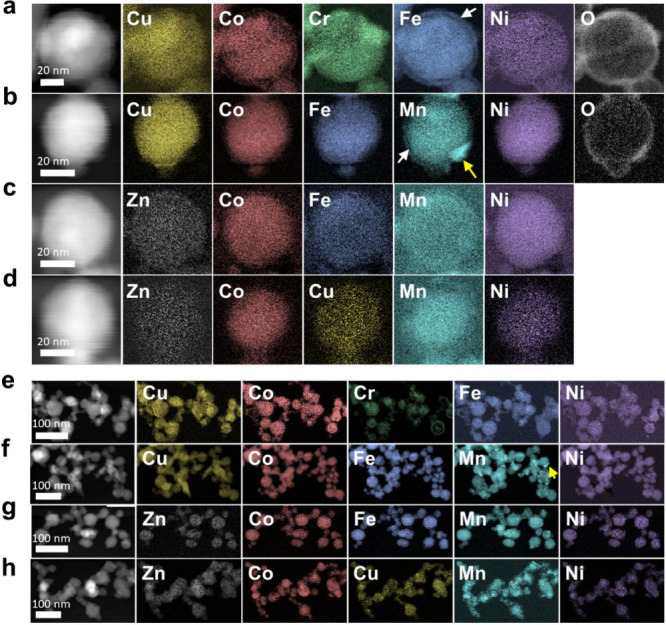
Elemental maps acquired using EELS under STEM mode. Single HEA NP: (a) CrFeCoNiCu HEA NP; (b) MnFeCoNiCu HEA NPs; (c) MnFeCoNiZn HEA NPs; (d) MnCoNiCuZn HEA NPs. Scale bar, 20 nm. Multiple HEA NPs: (e) CrFeCoNiCu HEA NP; (f) MnFeCoNiCu HEA NPs; (g) MnFeCoNiZn HEA NPs; (h) MnCoNiCuZn HEA NPs. Scale bar, 100 nm.

In our previous synthesis of CrFeCoNiCu HEA NPs via ICPJ,^23^ Cu tended to segregate to the particle surface, leading to the formation of core–shell type NPs while Cr, Fe, Co, and Ni were evenly distributed across the NP volume (see Cu elemental map in Figure 1b). Such segregation is driven by the tendency of Cu to minimize its interaction with other elements by diffusing toward the surface rather than remaining in the bulk. Interestingly, the core–shell-like Cu segregation is not observable in the current CrFeCoNiCu HEA sample produced with the nozzle (Figure 3a), suggesting that both the increased cooling rate and short residence time achieved by the nozzle may effectively limit the Cu diffusion upon molten state.

A statistical quantitative analysis was performed using TEM-EDX to evaluate the compositions of each HEA NP sample produced with the nozzle (Figure S10). The average compositions are estimated as Cr (18.6%), Fe (18.4%), Co (22.8%), Ni (24.6%), and Cu (15.6%) for CrFeCoNiCu HEA NPs; Mn (19.5%), Fe (22.8%), Co (23.0%), Ni (21.4%), and Cu (13.4%) for MnFeCoNiCu HEA NPs; Mn (20.9%), Fe (26.4%), Co (25.6%), Ni (22.5%), and Zn (4.5%) for MnFeCoNiZn HEA NPs; Mn (23.7%), Co (21.4%), Ni (21.3%), Cu (23.6%), and Zn (9.9%) for MnCoNiCuZn HEA NPs, respectively. It can be seen that the elemental percentages of the HEA NPs differ from those of the feedstock (i.e., equimolar ratio), especially for Zn. This can be rationalized by its relatively low boiling point (1180 K) and more critically, its high vapor pressure (7.83 × 10^5^ Pa at 1400 K) compared with the other elements (Cr, 6.74 × 10^–3^ Pa; Mn, 24.9 Pa; Fe, 1.6 × 10^–3^ Pa; Co, 4.19 × 10^–4^ Pa; Ni, 4.71 × 10^–4^ Pa; and Cu, 0.135 Pa).^38^ Because of its high vapor pressure, zinc requires a lower temperature to reach a supersaturation level comparable to that of other elements. During the ultrafast quenching process achieved by the supersonic nozzle, the time window available for Zn condensation is extremely short. Consequently, the condensation efficiency decreases, as a portion of Zn remains in the vapor phase for a longer time and may not fully condense before solidification. Furthermore, Zn atoms can undergo re-evaporation due to their high volatility during the early stage of particle growth. These combined effects lead to reduced incorporation of Zn into the final NPs. For the same reasons, Cu also shows low concentrations in the CrFeCoNiCu and MnFeCoNiCu HEA, in agreement with the trends reported in the HEA NP synthesis via aerosol or carbo-thermal shock (CTS) methods.^4,11^ A common approach to achieve targeted atomic ratios of the volatile elements like Cu and Zn is to increase their fractions in the initial feedstock accordingly.

### Phase Stability and Lattice Constants by DFT

DFT simulations were performed to investigate the phase stability and the lattice constants of the HEA NPs synthesized with the nozzle. In the DFT simulations, the supercells of each HEA system contain 125 atoms with the element ratios identified from the TEM-EDX analysis: Cr_23_Fe_23_Co_29_Ni_31_Cu_19_; Mn_24_Fe_28_Co_29_Ni_27_Cu_17_; Mn_26_Fe_33_Co_32_Ni_28_Zn_6_; Mn_30_Co_27_Ni_27_Cu_29_Zn_12_. The estimated valence electron concentration (VEC) values (VEC > 8.0) predict FCC structures for all the compositions in their ground states. Although the precise magnetic ground states of the FCC HEAs are not determined, both nonmagnetic (NM) and ferromagnetic (FM) configurations were considered in the DFT simulations. The total energies of the four FCC HEAs were calculated as a function of lattice parameter for both NM and FM states, as shown in Figure 4, from which the corresponding lattice constants were obtained. The results indicate that the FM states are more stable than the NM states. For all HEAs, the NM lattice constants are consistently lower compared to the FM values, with an average difference of 0.054 Å. The calculated lattice constants were compared with the values estimated from Vegard’s law and measured by XRD (Figure 4b), as also summarized in Table S5. For Vegard’s law, the lattice constants of the pure elements were taken from the Materials Project database to ensure theoretical consistency. Among all cases, the experimental XRD values are the largest, likely due to the presence of defects in the samples. The average deviations from the experimental values are 0.55% for FM-DFT, 2.06% for NM-DFT, and 1.04% for Vegard’s law, indicating that FM-DFT calculations provide the closest agreement with the XRD measurements.

**Figure 4:**
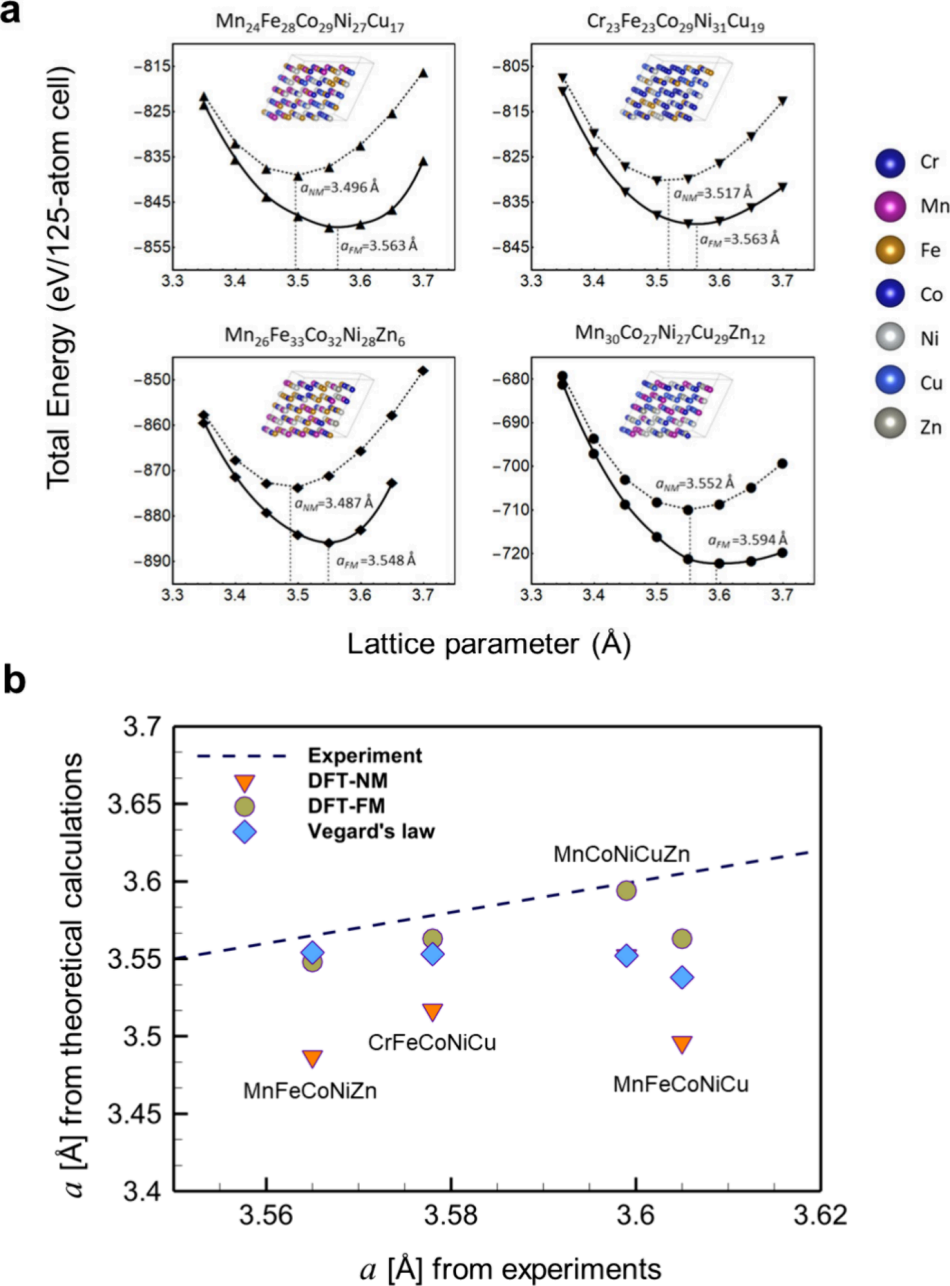
DFT simulation. (a) Total energy of FCC HEA as a function of lattice parameter in the nonmagnetic (dashed line) and ferromagnetic (solid line) states for Mn_24_Fe_28_Co_29_Ni_27_Cu_17_, Cr_23_Fe_23_Co_29_Ni_31_Cu_19_, Mn_26_Fe_33_Co_32_Ni_28_Zn_6_, and Mn_30_Co_27_Ni_27_Cu_29_Zn_12_. (b) Comparison of theoretical and experimental lattice constants (*a*) of the HEA NPs synthesized with the nozzle.

### Effects of the CD Nozzle

The temperature and velocity distributions calculated without and with the nozzle are shown in Figure 5a and Figure 5b, respectively. In both cases, the average temperature inside the torch is well above 5000 K, ensuring the near complete vaporization of the feedstock, including refractory metal precursors. However, the simulation result also indicates a reduced heating zone with the nozzle which may lead to incomplete vaporization of feedstock (see Figures S11 and S12). By the supersonic expansion (see Figure S13 for the static pressure profile), the nozzle drastically increases the plasma velocity up to Mach number *M*_a_ ≈ 1.5 from the torch exit as shown in Figure 5c. This can substantially affect the thermal history of the precursors and particles. Figure 5d and Figure 5e depict the axial profiles of temperature and heating/cooling rate calculated without and with the nozzle, respectively. Notably, with the nozzle, the maximum cooling rate increases from ∼10^6^ K/s to ∼10^8^ K/s. During the synthesis of CrFeCoNiCu samples, optical emission spectroscopy (OES) measurement was performed at *Z* = 0.49 m from the top of the plasma torch, both with and without the nozzle (see Figure S14). Without the nozzle, intense emission from elemental metal vapors was observed, consistent with the high temperature (4215 K) and the extended high-temperature zone predicted by the CFD simulation. In contrast, with the nozzle, the emission intensity decreased, which may associate with the metal vapor consumption through nucleation and co-condensation processes, as well as changes in local temperature, excitation efficiency, and plasma density. The calculated temperature (2050 K) is close to the estimated nucleation temperature (e.g., nickel at 1776 K, Figure 5f,g), providing indirect validation of the simulation results.

**Figure 5:**
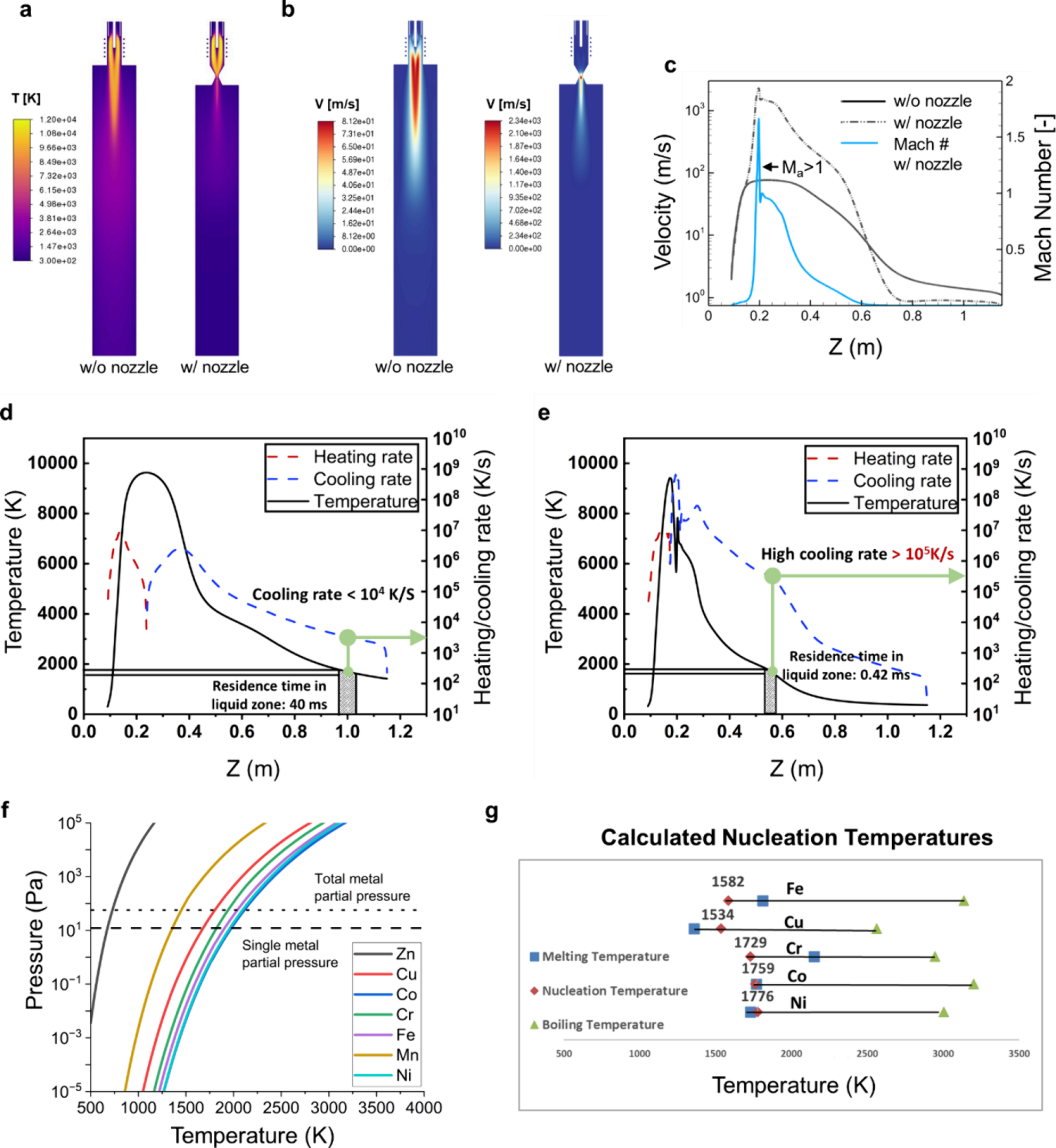
Thermofluid simulations, homogeneous nucleation calculations and effect of supersonic nozzle on HEA NPs growth. (a) Temperature distributions inside the reactor calculated with and without the supersonic nozzle. (b) Velocity distributions inside the reactor calculated with and without the supersonic nozzle. (c) Axial velocity profiles and Mach numbers calculated with and without the supersonic nozzle. Axial temperature profiles with local heating and cooling rates calculated for (d) with and (e) without the supersonic nozzle. The shaded areas represent the estimated liquid zones. Calculated (f) saturation vapor pressures and (g) nucleation temperatures of each element in a vapor mix of Co:Cr:Cu:Fe:Ni = 1:1:1:1:1 produced at a feed rate of 0.5 g/min, showing the existence of a nucleation temperature gap.

The liquid zone (defined as the range between the nucleation temperature of the first-nucleating element and the solidification temperature of the resulting HEA alloy) is critical for controlling of HEA NP formation. Within this zone, the element having the highest nucleation temperature (e.g., nickel at 1776 K for the Cr:Co:Fe:Ni:Cu = 1:1:1:1:1 case, Figure 5f,g) forms nuclei first via homogeneous nucleation,^39,40^ and then the nuclei continue to grow to HEA NPs by incorporating metal monomers from the multicomponent vapor via the cocondensation process. Assuming the molten state in this zone, elements inside particles can interdiffuse and undergo either alloying or segregation process. As such, the residence time and thermal history in the liquid zone should in principle controls both particle growth (via cocondensation and coagulation) and their chemical homogeneity (via element diffusion).

For the CrFeCoNiCu HEA NP sample, the substantial configurational entropy (13.31 J/mol) indicates the formation of a solid-solution at its melting temperature (see Table S6).^41^ However, due to the high interatomic potential energy of Cu and the large positive binary mixing enthalpies of Cu–Cr (12 kJ/mol), Cu–Fe (13 kJ/mol), and Cu–Co (6 kJ/mol),^42^ it may result in Cu segregation during the liquid phase as reported in our previous work.^23^ The melting temperature of this HEA NP, adjusted for nanoscale effects, is estimated to be 1651 K.^43,44^ The corresponding liquid zones are indicated in Figure 5d,e for the cases without and with the nozzle, respectively. The residence time in the liquid zone, extracted from the corresponding temperature gradient and cooling rate, shows a substantial reduction from 40 to 0.42 ms when the nozzle is employed. Such ultrafast cooling and short residence time can significantly limit particle growth and element diffusion, effectively “freezing out” their well-mixed state at high temperature into a uniform solid-solution. This simulation result well supports our experimental observations.

We quantify the kinetical trapping mechanism by comparing the residence time in the liquid-phase (i.e., 0.42 ms) to the characteristic time required for Cu diffusion toward the particle surface. The diffusion of Cu in a CrFeCoNi-based alloy is assumed to follow an Arrhenius equation:^45^

$$D_{V}^{\mathrm{Cu}}=,\left(6.6_{-5.0}^{+19.5}\right)\times 10^{-10}\cdot \exp\left(-\frac{\left(149.9\pm 12.1\right)\mathrm{kJ}/\mathrm{mol}}{RT}\right)m^{2}/s$$

where *R* is the gas constant and *T* is the temperature. Using literature values for the solute diffusion of Cu,^45^ the volume diffusion coefficient at the representative liquid-phase temperature of 1700 K is calculated to be 1.7 × 10^–14^ m^2^/s. For a 30 nm nanoparticle, the time required for a Cu atom to diffuse from the core to the surface is approximately 13 ms based on random-walk theory (*r* ^2^ ∝ *Dt*). This estimate is an order of magnitude longer than the residence time of 0.42 ms achieved by the nozzle, providing a good explanation for why it is unlikely for Cu to segregate before the nanoparticle solidifies. Conversely, without the nozzle, the residence time of 40 ms is ample for segregation to occur, consistent with our experimental observations. Under equilibrium conditions, smaller particles typically exhibit enhanced surface segregation due to shorter diffusion distances to the surface and higher surface-to-volume ratios. However, the calculation indicates that, in the presence of the nozzle, the system operates in a kinetically limited regime, where solidification occurs faster than the diffusion time required for element segregation. Therefore, the homogeneous elemental distribution established at high temperature is effectively preserved, despite the reduced particle size. Despite the high binary mixing enthalpies of Cu–Cr (12 kJ/mol), Cu–Fe (13 kJ/mol), Cu–Co (6 kJ/mol), and Zn–Fe (4 kJ/mol), all other three HEA samples, MnFeCoNiCu, MnFeCoNiZn, and MnCoNiCuZn, also exhibit homogeneous mixing of their constituent elements for the same reason. This result highlights the effectiveness and generalizability of the supersonic-nozzle-assisted plasma jet technique in producing fine, uniformly alloyed HEA NPs, even in the presence of miscibility gaps among constituent elements.

The synthesis of HEA NPs via the supersonic nozzle also results in a refinement of both particle morphology and internal crystal structure. SEM and XRD analyses in Tables S2 and S3 reveal that the average particle size is reduced from 130 to 30 nm, while the mean crystallite size decreases from 17 to 11 nm (∼40%) upon utilizing the supersonic nozzle. Further microstructural analysis shows that the ultrafast cooling rate achieved by the nozzle alters the formation of twin structures. Figure 6a shows a CrFeCoNiCu HEA NPs synthesized without the nozzle, which is finely laminated with nanotwins (NTs) exhibiting uniform thickness and a narrow twinning-width distribution. This indicates that the relatively “stable” thermal history experienced by the nanoparticles provided sufficient time for the NTs to develop fully. An enlarged view of a similar region prepared by FIB is shown in Figure 6b, revealing the detailed twinning structure. Two Burgers circuits, labeled I and II, are drawn on the Fourier-filtered image. Two Shockley partial dislocations along the ⟨112⟩ direction are marked by red arrows. The twin plane is identified as the {111} close-packed plane, as indicated in Figure 6b. In contrast, the NTs within the CrFeCoNiCu HEA NP synthesized with the nozzle exhibit a broader twinning-width distribution (Figure 6c). This behavior can be attributed to heterogeneous nucleation and growth of twins induced by deformation within the nanoparticle, which originates from internal stresses generated by the ultrafast cooling rate associated with the nozzle-assisted synthesis process.^46^ Furthermore, the 9R-phase-like modulated structure observed within the NTs (regions indicated by arrows in Figure 6d) provides additional support for this interpretation.^47^

**Figure 6:**
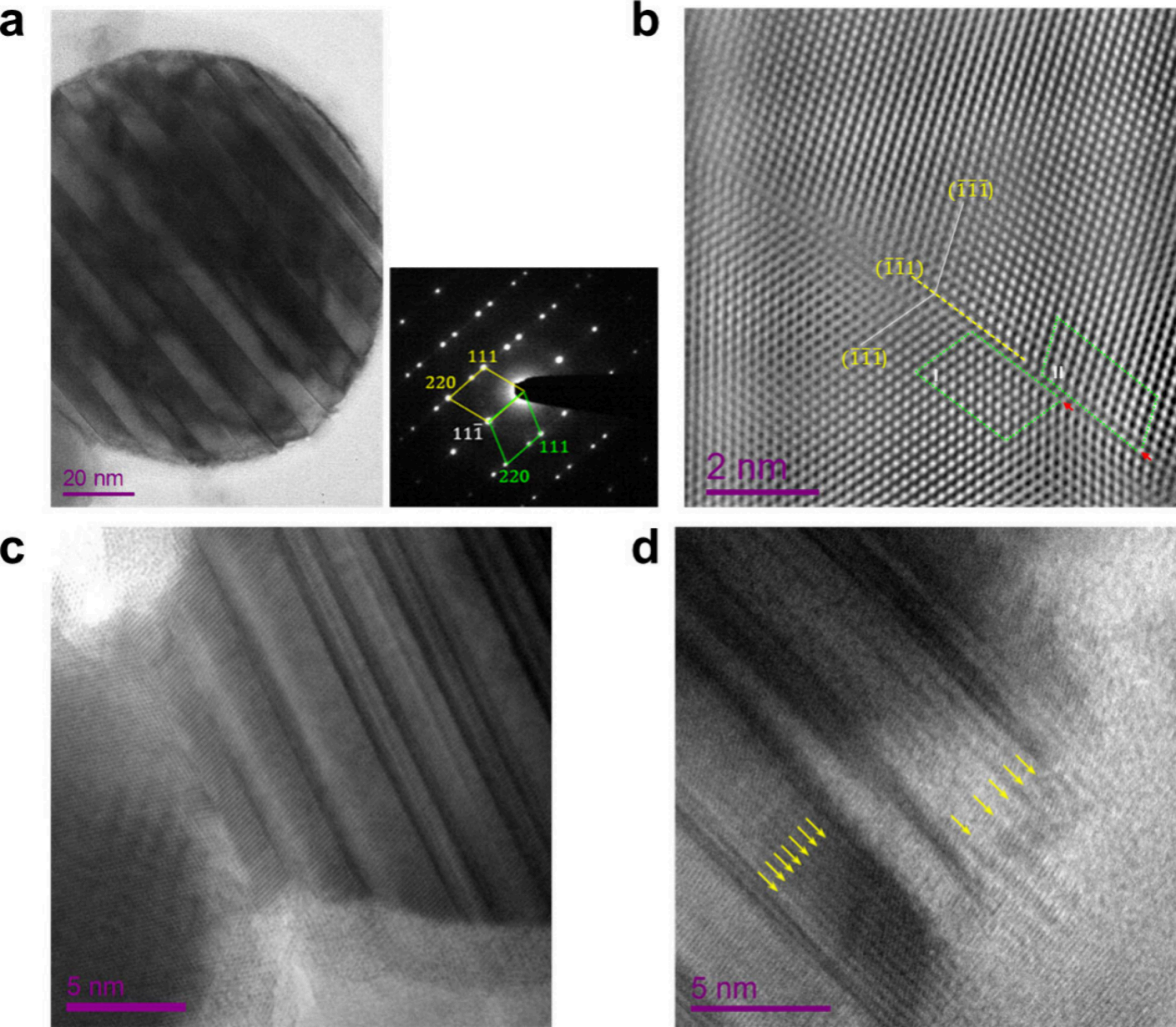
(a) Nanotwins (NTs) in a CrFeCoNiCu HEA nanoparticle synthesized without the nozzle, together with the corresponding SAED pattern. (b) High-resolution TEM (HRTEM) image of the NTs, showing two Shockley partial dislocations indicated by red arrows. (c) Nanotwins formed in a CrFeCoNiCu HEA nanoparticle synthesized with the nozzle. (d) Enlarged view of NTs in another region, highlighting modulated structures within the NTs, as indicated by arrows.

Figure 7 schematically summarizes the profound, multifaceted effect of the supersonic flow on the growth of HEA NPs by a thermal plasma jet, providing ultrafast quenching that increases the maximum cooling rate from ∼10^6^ to ∼10^8^ K/s and drastically reduces the residence time in the liquid phase from 40 ms to less than half a millisecond (0.42 ms). First, it kinetically limits particle growth, resulting in smaller particles (∼30 nm) with a narrow size distribution compared to those produced without the nozzle (∼100 nm). Second, it limits atomic diffusion, effectively “freezing” the elements into a homogeneous solid solution and preventing elemental segregation observed in the slowly cooled particles. Finally, it shifts the solidification pathway toward a more rapidly quenched growth regime, evidenced by lattice parameter contraction (∼0.01 Å), crystallite size reduction (∼40%) and formation of nonuniform NT lamellae. We acknowledge that the average particle size (∼30 nm) is still larger than that achieved by some alternative approaches; however, it is comparable to other scalable techniques and provides a favorable balance among particle size, compositional homogeneity, and production rate. In addition, this plasma method enables the direct use of pure elemental metal feedstocks for the continuous synthesis of HEA NPs. A comparison table in Supporting Information summarizes the advantages and limitations of this plasma process relative to other HEA NP synthesis methods (Table S7).

**Figure 7:**
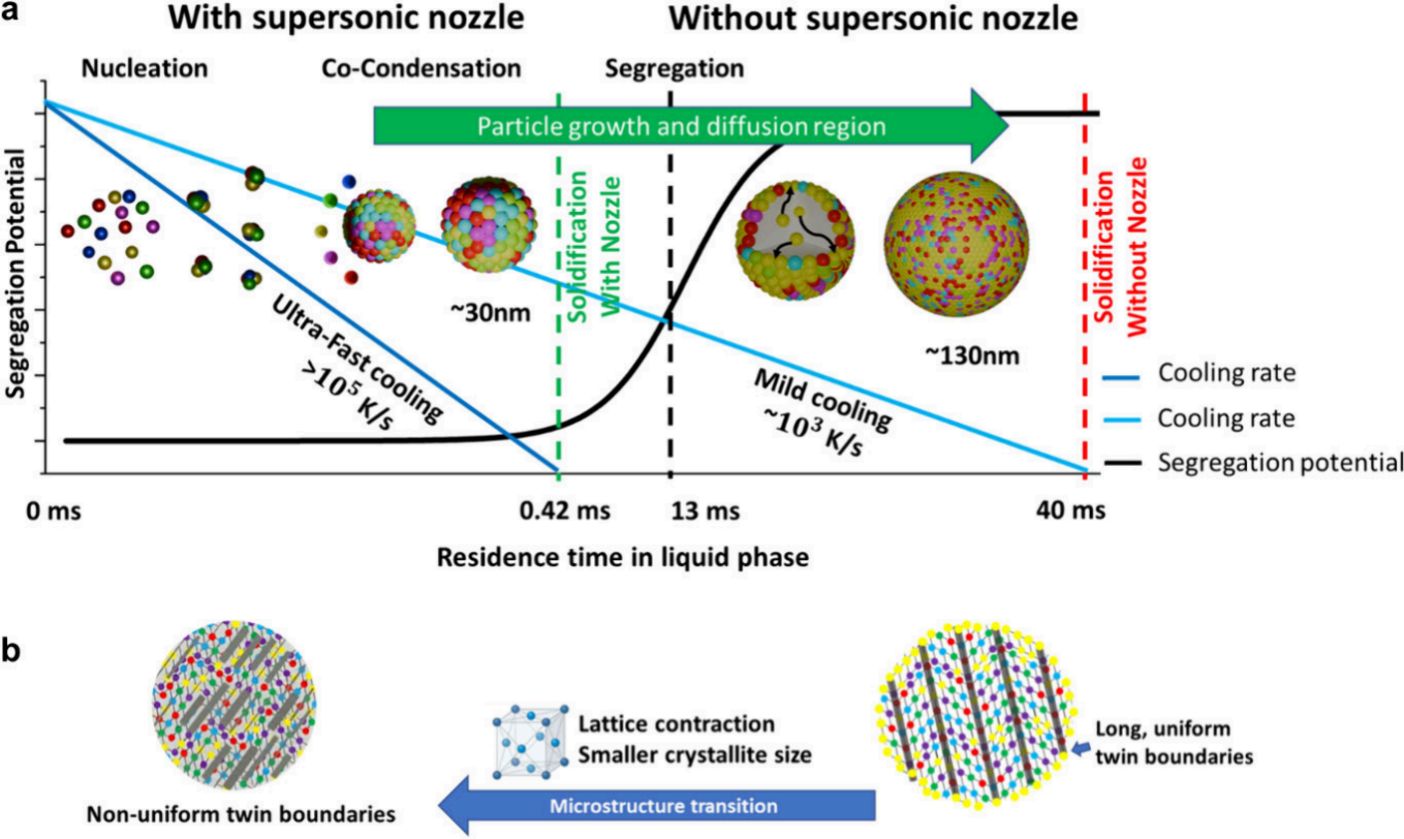
Schematic illustration of effects of the supersonic nozzle on the growth of HEA NPs via the ICPJ process. (a) Morphology and compositional uniformity. The supersonic nozzle provides ultrafast cooling (>10^5^ K/s), which limits both particle growth and elemental segregation, leading to formation of uniformly mixed, small-sized HEA NPs (∼30 nm). (b) Evolution of microstructure. The high cooling rate promotes lattice contraction and formation of nonuniform twin lamellae at nanoscales. The schematic is not to scale and is simply intended to illustrate qualitative trends in the cooling rate, diffusion rate, growth kinetics, and microstructure evolution.

## Conclusion

A supersonic-nozzle-assisted thermal plasma process is demonstrated for the continuous synthesis of compositionally uniform HEA NPs at scales. The approach enables ultrafast cooling rates (10^6^–10^8^ K/s) with highly directional flow, preventing elemental segregation and producing uniform, single-phase HEA NPs with average sizes around 30 nm at a high production rate of ∼5 g/h. CFD simulations reveal that the ultrafast cooling rate significantly reduces the residence time in the liquid phase zone to a submillisecond time scale (0.42 ms). Quantitative diffusion calculations confirm that the residence time calculated is short enough to kinetically limit atomic diffusion, preventing the segregation of elements such as Cu. A detailed study of the microstructure also elucidates the deterministic role of the cooling rate in the growth of NPs with different internal structures. Rapid solidification leads to a slight lattice contraction and a ∼40 % reduction in the coherent diffraction domain size due to enhanced lattice strains and formation of NT boundaries. Concomitantly, the twin structure evolves from long-range, uniform twin lamellae to nonuniform ones that are characteristic of a rapid, nonequilibrium solidification pathway.

The contribution of this study goes beyond addressing the limitations of our prior work through nozzle design. More importantly, it demonstrates kinetically controlled synthesis of HEA NPs, enabling reduced particle size, suppression of elemental diffusion, and microstructural evolution toward characteristics of rapid solidification. CFD modeling and extensive characterization also provide mechanistic insights into nucleation and solidification behavior of HEA NPs by correlating the achieved flow structure with thermal history and residence time of a particle. This work establishes a generalizable framework for developing nonequilibrium synthesis routes for complex nanomaterial systems by high-temperature plasmas. While the present study focuses on synthesis and structural characterization, the HEA NPs produced by this method could be promising candidates for catalytic and energy-related applications. The combination of compositional uniformity, tunable microstructure, and scalable production offers new opportunities to optimize functional properties for practical use. Moreover, metal–oxide core–shell structure observed in this study could enhance catalytic activity and stability. Future work will include application-specific testing and surface engineering to further explore these potentials.

## Experimental Section

### Chemicals and Materials

To synthesize HEA NPs with four different compositions (i.e., CrFeCoNiCu, MnFeCoNiCu, MnFeCoNiZn, and MnCoNiCuZn), the feedstock powder including copper (Cu, 0.5–1.5 μm, 99%), chromium (Cr, <10 μm, 99.2%), iron (Fe, 6–10 μm, 99.5%), cobalt (Co, 1.6 μm, 99.8%), nickel (Ni, <50 μm, 99.7%), manganese (Mn, <10 μm, 99.6%), and zinc (7.5 μm, 98.8%) was purchased from MillporeSigma and Thermo Fisher. The as-received metal powders were mixed at an equal molar ratio (1:1:1:1:1) for 7 h using a powder mixer prior to synthesis experiments without further treatment.

### Synthesis of HEA NPs with a Supersonic Nozzle

The synthesis system comprises five main components: an induction plasma torch (Tekna PS-50, 45 kW, ∼3 MHz), a supersonic nozzle, a reaction chamber, a cyclone separator, and a filtration unit (Figure S2). During each synthesis experiment, the feedstock mixture was continuously introduced into an Ar–H_2_ (8.3%) plasma at a feed rate ranging from 0.5 to 1 g/min using a vibrating powder feeder (PFR200 feeder, Tekna Systems, Inc.). The plasma gas composition employed was 5 slpm of carrier gas (Ar), 30 slpm of central gas (Ar), and 120/14 slpm of sheath gas (Ar/H_2_). The typical run lasted for an hour and the reactor pressure was maintained at 22.67 kPa. All resulting powders in filtration chamber and cyclone chamber were collected and handled in a nitrogen filled glovebox. From an initial 30 g of feedstock, approximately 3 and 5 g (∼20% yield) of powder were collected from the cyclone and filter, respectively, with the remaining material lost primarily to wall deposition within the reaction chamber, which could be minimized by switching gas-flowing porous reactor walls. During the synthesis experiments, optical emission spectra were measured at *Z* = 0.49 m from the top of the plasma torch. A modular spectrometer (JAZ-EL200-XR1, Ocean Optics, with 1.7 nm fwhm resolution) was used with a wavelength range from 200 to 1025 nm.

### Materials Characterization

#### XRD

The as-produced samples were loaded and measured on an acrylic sample holder. X-ray diffraction (XRD) patterns were obtained on a diffractometer Bruker D8 Advance II with monochromatized Cu Kα radiation (λ = 1.5406 Å, 40 kV, 40 mA) at a scanning rate of 1.2°/min. The scanned range was 10°–90°.

#### SEM/STEM Imaging and Analysis

Two scanning electron microscopy (SEM) systems were employed for the morphological and compositional characterization of HEA NPs. First, a Hitachi S-5500 cold field-emission SEM operated in scanning transmission electron microscopy (STEM) mode was used for high-resolution imaging of nanoparticles. HEA samples (0.1 g) were suspended in 100 mL of water (0.1% w/v) and agitated for 1 min to form a colloid. A droplet was mounted on a carbon-coated 400-mesh copper grid, blotted after 30 s, and air-dried overnight before SEM/STEM analysis. SEM/STEM images were obtained at 30 kV, 30 μA emission current. Length and width of well-separated HEA NPs (total count of 500) were analyzed using the ImageJ software (National Institutes of Health, Rockville, MD, USA). Energy-dispersive X-ray spectroscopy (EDX) mapping was performed at 30 kV and 30 μA for 10 min to identify the elemental composition across the samples, using a Bruker XFlash 5030 EDX detector.

Second, for the statistical analysis on the surface morphology and bulk composition, a cold field-emission SEM (Hitachi S-4800) equipped with an Oxford Instruments Aztec Ultim Max 170 EDX detector was used. Sample powders were attached to a conductive glue tape. SEM imaging was performed in secondary electron mode at 3.5 kV accelerating voltage. EDX was performed in two modes: low resolution mode at 15–20 kV and high-resolution mode at 3.5–5.0 kV. Conventional EDX elemental analysis (low resolution mode) performed at 15–20 kV had interaction volume (the volume where the electron beam reaches and causes X-ray generation) in micrometer range, while at 3–5 kV, the interaction volume decreased to nanometer range. Barkshire et al. calculated spatial resolution of X-ray emission in Ni (L_α_ line) to be 24 nm at 5 kV and 18 nm at 3 kV.^48^ Spatial resolution of other elements with similar atomic number (such as Fe, Cu, Co, Mo, Mn) is expected to be in the same range and the resolution for elements with higher atomic number tends to be higher than those with lower atomic number.^48–50^

#### TEM

Two transmission electron microscopy (TEM) systems were employed. The first TEM set up was used mainly for the composition analysis performed using a FEI Titan3 80-300 operated at 300 kV and equipped with a CEOS aberration corrector for the probe-forming lens. TEM specimens were prepared by dispersing the solid powder in ethanol and sonicating for 5 min. A drop of the resulting suspension was deposited onto an ultrathin carbon film supported by a lacey carbon film on a 400-mesh gold grid and dried in air. Both high-resolution TEM (HRTEM) and annular dark-field (ADF) STEM images were acquired. ADF images were collected using a high-angle annular dark-field (HAADF) detector (Fischione). The instrument was also equipped with an EDX spectrometer (EDAX Analyzer, DPP-II) and an electron energy-loss spectrometer (Gatan Tridiem 866 image filter) for elemental analysis and mapping. To optimize the signal intensity, EDX spectra were acquired with the specimen tilted at 15°. EELS was performed using a Gatan Tridiem 866 imaging filter equipped with a CCD camera. Spatially resolved EELS spectrum images were acquired in STEM mode for elemental mapping. Elemental maps were generated by integrating ionization edges after background subtraction in EELS, and by integrating characteristic X-ray peaks in EDX.

The second TEM setup was used for microstructure characterization and defect analysis performed by a Hitachi H-9500 TEM at an accelerating voltage of 300 kV. The as-produced HEA NP powder was dispersed in DI water and sonicated in ultrasonic bath for 2 min. One or two drops of solution were directly placed onto a lacey carbon film coated copper grid (400 mesh, Electron Microscopy Sciences (EMS), USA) and let dry in the air overnight. FIB samples were prepared on a Hitachi NB5000 FIB-SEM (40 kV) by depositing a carbon frame and extracting roughly 10 μm × 10 μm regions to mount on 4 finger copper FIB grids. Particles of interest were subsequently thinned to approximately 100 nm using a 40 kV, 20 pA Ga+ beam cutting parallel to the surface of the extracted region for TEM imaging.

### Density Functional Theory (DFT) Calculations

Structural relaxation and total energy calculations were performed using density functional theory (DFT) as implemented in the Vienna ab initio simulation package (VASP). The projector augmented-wave (PAW) method and the generalized gradient approximation (GGA) with the Perdew–Burke–Ernzerhof (PBE) parametrization were employed.^51–53^ Face-centered cubic (FCC) HEA supercell models containing 125 atoms were constructed, with random solid-solution configurations generated using the hybrid Cuckoo Search (CS) algorithm.^54^ For each FCC HEA, the lattice constant was optimized by evaluating the total energy as a function of lattice parameter, varied from 3.35 Å to 3.70 Å in increments of 0.05 Å. At each lattice constant, the total energy was obtained after relaxing all atomic positions via the conjugate-gradient algorithm, while keeping both the FCC structure and lattice constant fixed. The plane-wave basis set employed a kinetic energy cutoff of 520 eV, and Brillouin zone sampling was performed using a 2 × 2 × 2 Monkhorst–Pack k-point grid. Spin-polarized calculations were carried out for FM states with collinear spins initialized in the all-spin-up configuration, whereas non-spin-polarized calculations were performed for the NM states.

### Nucleation Temperature Calculation and CFD Simulations

To understand the nucleation and growth of HEA NPs from the multicomponent metal vapors, nucleation temperature calculations were performed for different HEA compositions. 2D computation fluid dynamic (CFD) simulations were performed for a reactor with a supersonic nozzle to better understand the thermal and velocity fields inside the reactor. The plasma model was built based on the magneto-hydrodynamic (MHD) theory with assumptions widely adopted in thermal plasma modeling, including local thermodynamic equilibrium (LTE), a quasi-neutral optically thin plasma, and temperature-dependent thermophysical properties.^23,55^ Due to the complexity of multicomponent HEA systems, the injection of metal precursors and their phase transformation in the plasma jet (e.g., melting and vaporization) were not considered. Such particle loading could influence the plasma temperature profile and transport properties by heat exchange between particles and plasma and altering plasma composition, respectively. However, since the feed rate in this study is relatively low (0.5–1.0 g/min) compare to the plasma power and flow rate, the loading effect is assumed to be insignificant. To accurate estimate particle residence time in the liquid zone, modeling NP formation from the multicomponent vapor and tracking their trajectories would be necessary. Since precursor injection and subsequent NP formation were not considered in this work, it is assumed that particles closely follow the plasma flow with the same velocity; the thermal history of particle and the residence time were approximated based on the gas temperature and velocity. Given that particles are nanometric in size with negligible inertia, this assumption is reasonable in both subsonic and supersonic regimes.^26,56^ More details on the background of this study can be found elsewhere.^23,55^

## Supplementary Material



## Notes

Design of a converging–diverging supersonic nozzle based on quasi-one-dimensional compressible flow theory; schematics and photos of the plasma processing system; additional characterization data including SEM, TEM, XRD, TEM-EELS, TEM-EDX, SEM-EDX; calculation of lattice constants of the HEA NPs synthesized; calculation of mixing enthalpies and entropies of HEA NPs (PDF)
