# Supplementary material for: Kinetically Controlled Synthesis of Non-Noble Metal Based High-Entropy Alloy Nanoparticles via Supersonic-Nozzle-Assisted Thermal Plasma Jet
Source: ACS Nano. 2026 Jul 8;20(28):20016–28. doi: 10.1021/acsnano.6c02465 (PMC13394527; doi:10.1021/acsnano.6c02465)
Supplement: Supplementary file 1 [file nn6c02465_si_001.pdf]

Supporting Information

Kinetically-controlled Synthesis of Non-noble Metal based High-entropy Alloy Nanoparticles via Supersonic-Nozzle-Assisted Thermal Plasma Jet

Ziqi Tang[2], Martin Couillard[4], Jian Chen[1], Homin Shin[1], Olga Naboka[5], James Tordiff[1], Jae-Young Cho[1,6], Thomas Lacelle[1], Dean Ruth[1], Mark Plunkett[1], Michel Nganbe[2] and Keun Su Kim[1,2,3]*

[1]Quantum and Nanotechnologies Research Centre, National Research Council Canada, Ottawa, ON K1A 0R6, and Edmonton, AB T6G 2M9, Canada

[2]Department of Mechanical Engineering, University of Ottawa, Ottawa, ON, K1N 6N5, Canada

[3]Department of Mechanical and Industrial Engineering, University of Toronto, Toronto, ON M5S 3G8, Canada

[4]Clean Energy Innovation Research Centre, National Research Council Canada, Ottawa, ON K1A 0R6, Canada

[5]Construction Research Centre, National Research Council Canada, Ottawa, ON K1A 0R6, Canada

[6]Department of Mechanical Engineering, University of Alberta, Edmonton, AB, T6G 1H9, Canada

KEYWORDS: *High-Entropy alloy nanoparticles, Thermal plasma, Supersonic nozzle, Ultrafast quenching, Kinetically-controlled synthesis*

[*]Corresponding author: Keun Su Kim; Tel: +1-613-998-5365; Fax: +1-613-991-2648

E-mail address: KeunSu.Kim@nrc-cnrc.gc.ca (K. S. Kim)

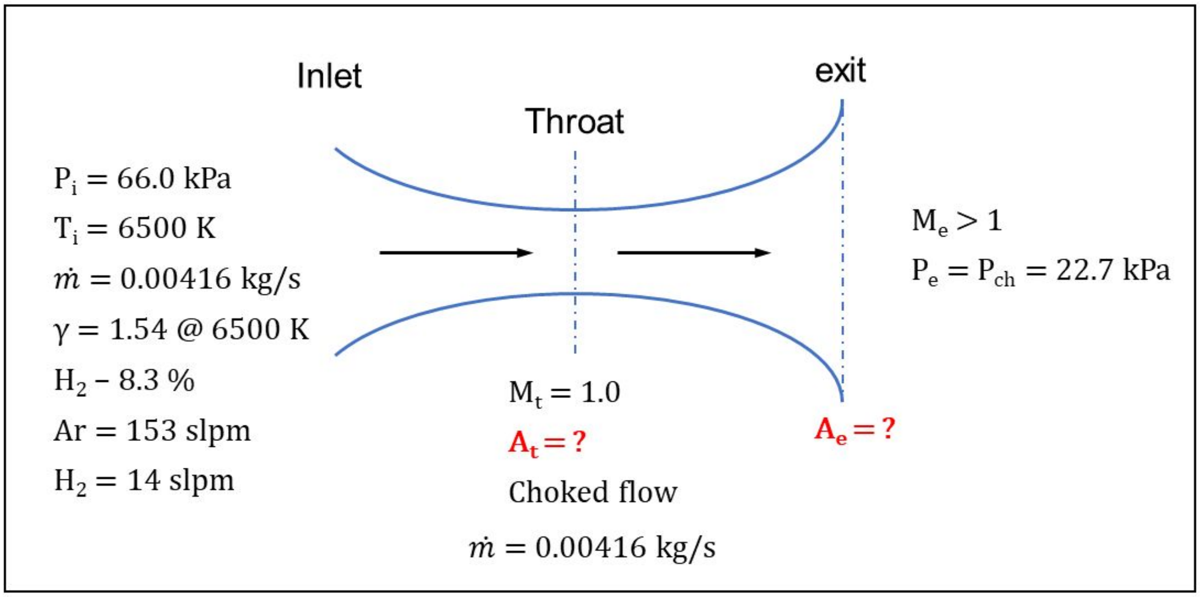

Figure S1. Design of a converging-diverging nozzle based on quasi-one-dimensional analysis.

- Mach #: $M_e = \sqrt{\frac{2}{\gamma\text{-}1}\left[\left(\frac{P_i}{P_e}\right)^{\frac{\gamma-1}{\gamma}} - 1\right]} = 1.30$

- Throat area: $A_t = \frac{\dot{m}}{P_i}\frac{\sqrt{T_i}}{\sqrt{\frac{\gamma}{R_g}\left(\frac{2}{\gamma+1}\right)^{\frac{\gamma+1}{\gamma-1}}}} = 0.0001079\ \text{m}^2$

- Exit area: $A_e = \frac{A_t}{M_e}\left[\left(\frac{2}{\gamma+1}\right)\left(1+\frac{\gamma-1}{2}M_e^2\right)\right]^{\frac{\gamma+1}{2(\gamma-1)}} = 0.000144\ \text{m}^2$

- $D_t = 0.1173$ m; $D_e = 0.01207$ m

where P_i is the inlet pressure, T_i is the inlet temperature, $\dot{m}$ is the mass flow rate, γ is the specific heat ratio, R_g is the gas constant, A_t is the throat area, D_t is the throat diameter, M_t is the Mach number at the throat, P_e is the exit pressure, P_{ch} is the chamber pressure, A_e is the exit area, D_e is the exit diameter, and M_e is the Mach number at the exit.

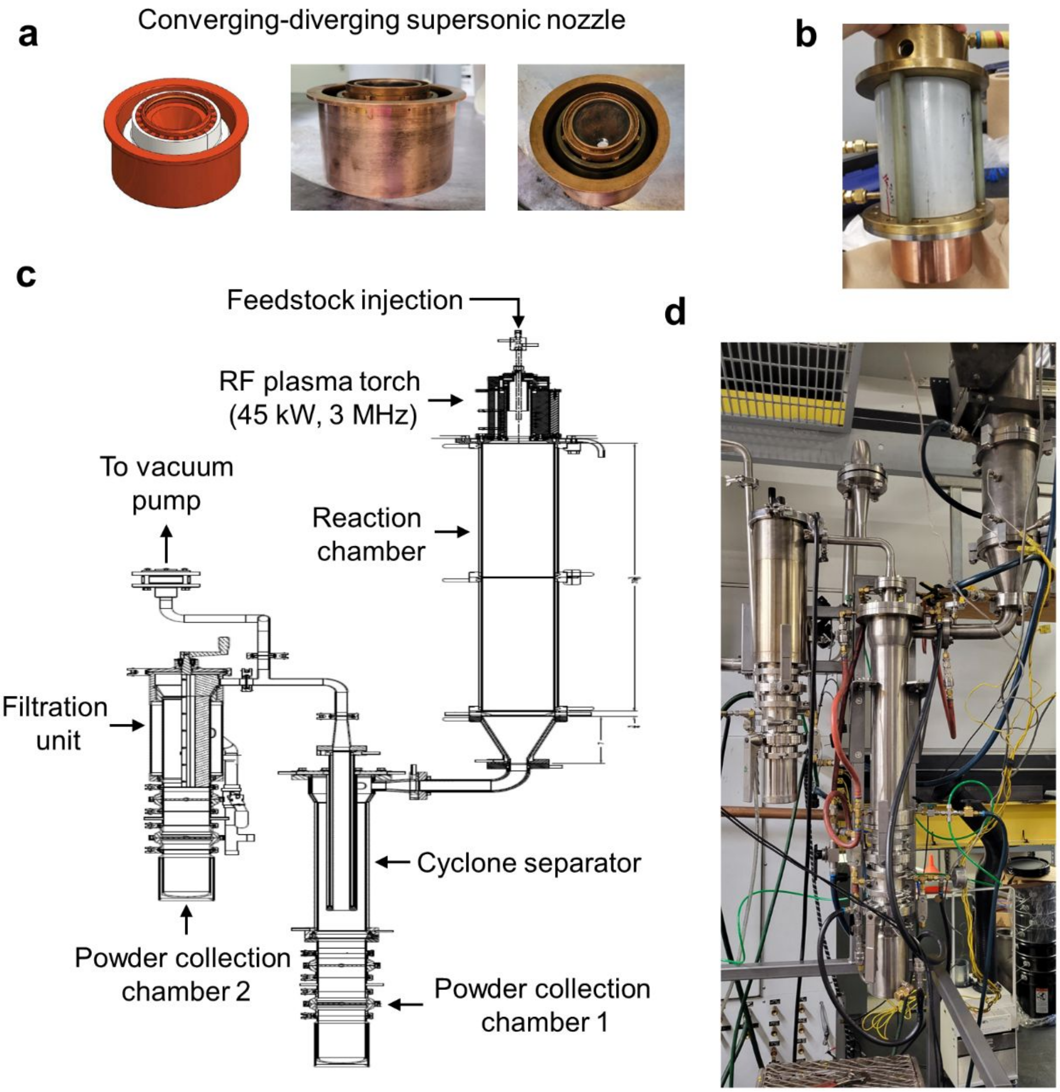

Figure S2. (a) Schematic and photos of the converging-diverging supersonic nozzle designed and fabricated. (b) Photo of the RF plasma torch with the nozzle. (c) Schematic of the plasma processing system employed for the synthesis of HEA NPs. (d) Photo of the plasma processing system.

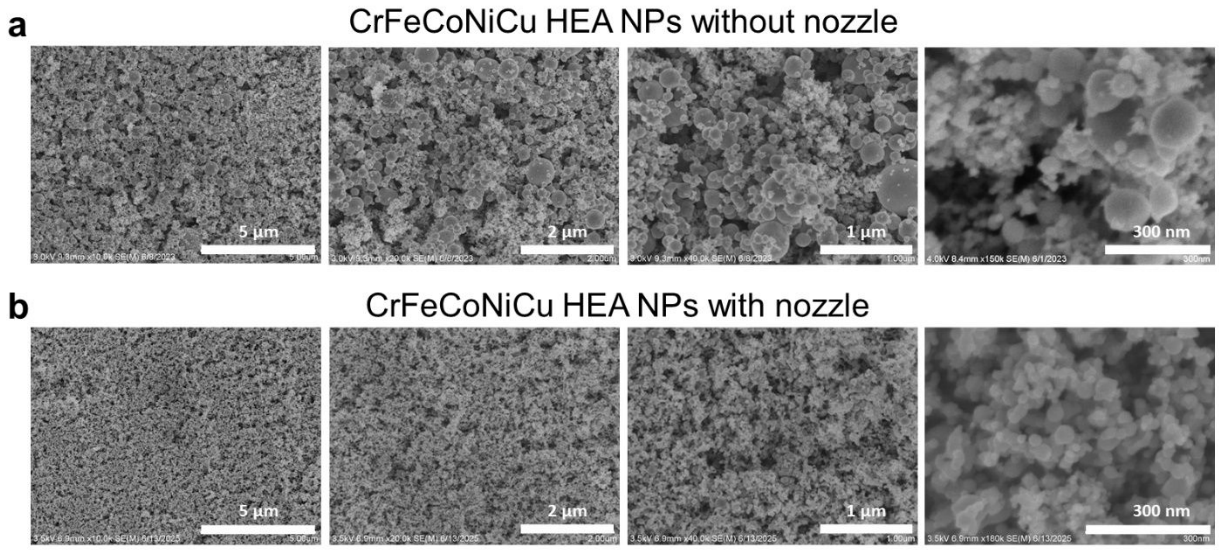

Figure S3. SEM images of the as-produced CrFeCoNiCu HEA NPs. (a) without and (b) with the nozzle.

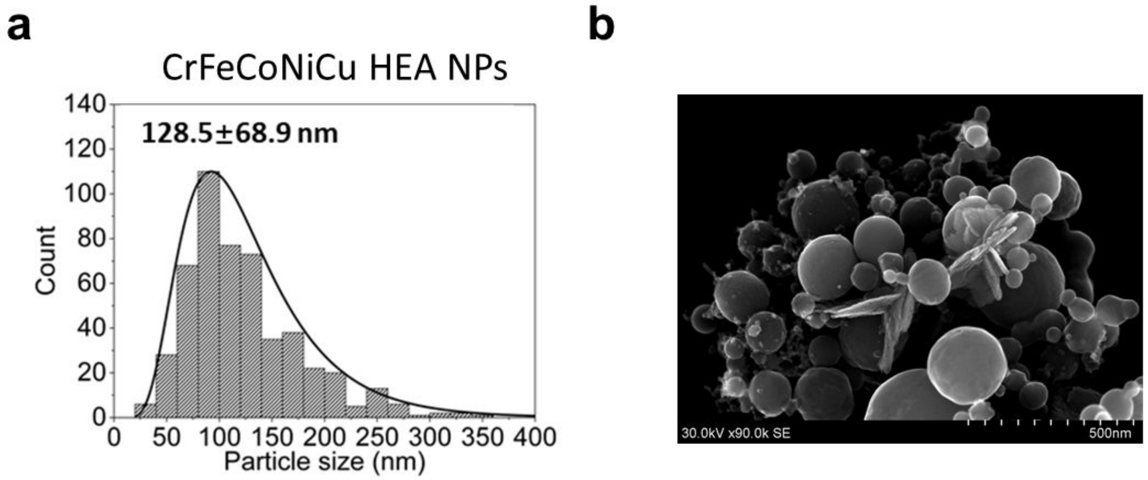

Figure S4. (a) Particle size distribution of the as-produced CrFeCoNiCu HEA NPs without the nozzle. (b) A SEM image of the as-produced CrFeCoNiCu HEA NPs.

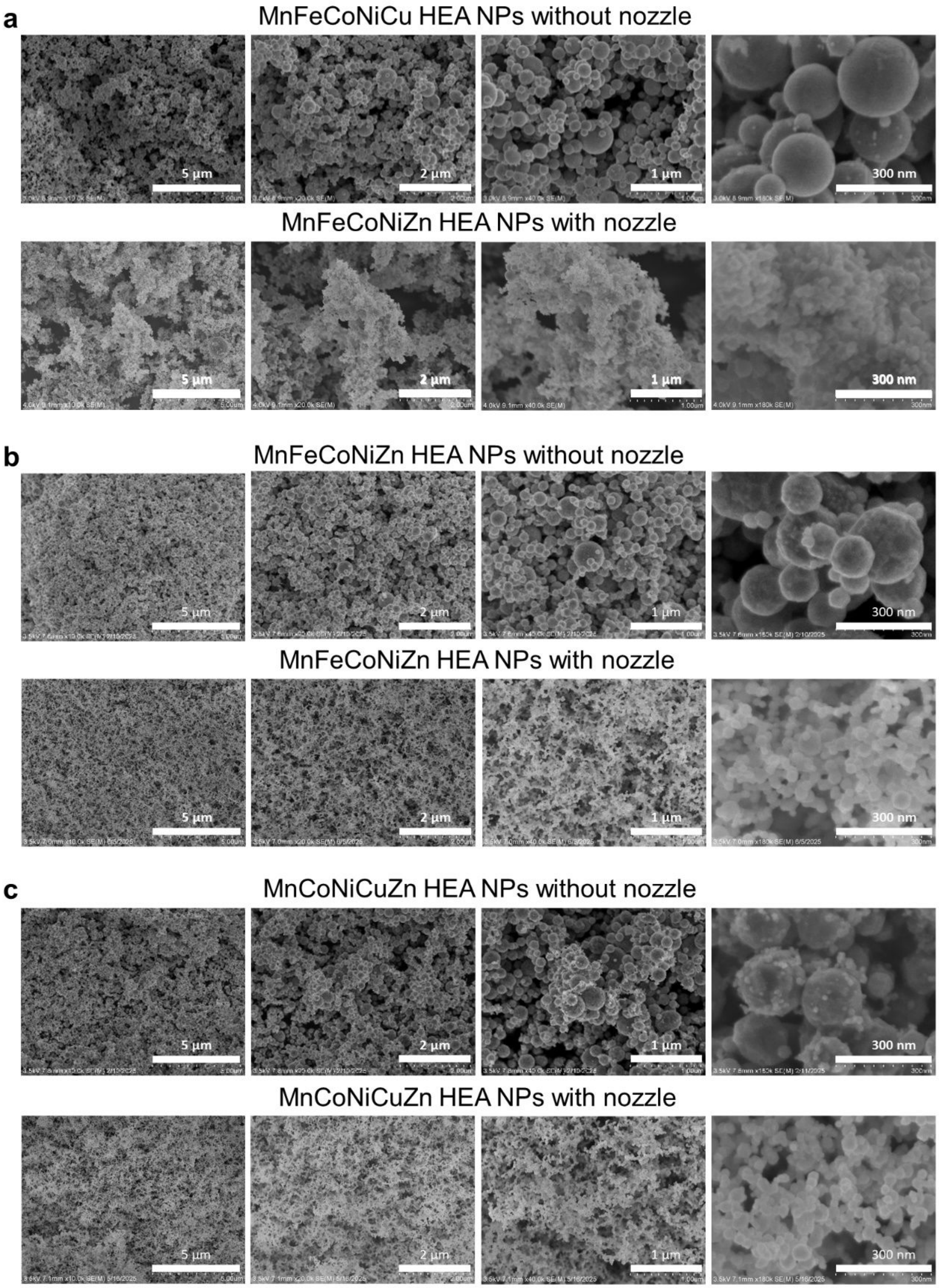

Figure S5. SEM images of the as-produced HEA NPs without and with the nozzle. (a) MnFeCoNiCu HEA NPs. (b) MnFeCoNiZn HEA NPs. (c) MnCoNiCuZn HEA NPs.

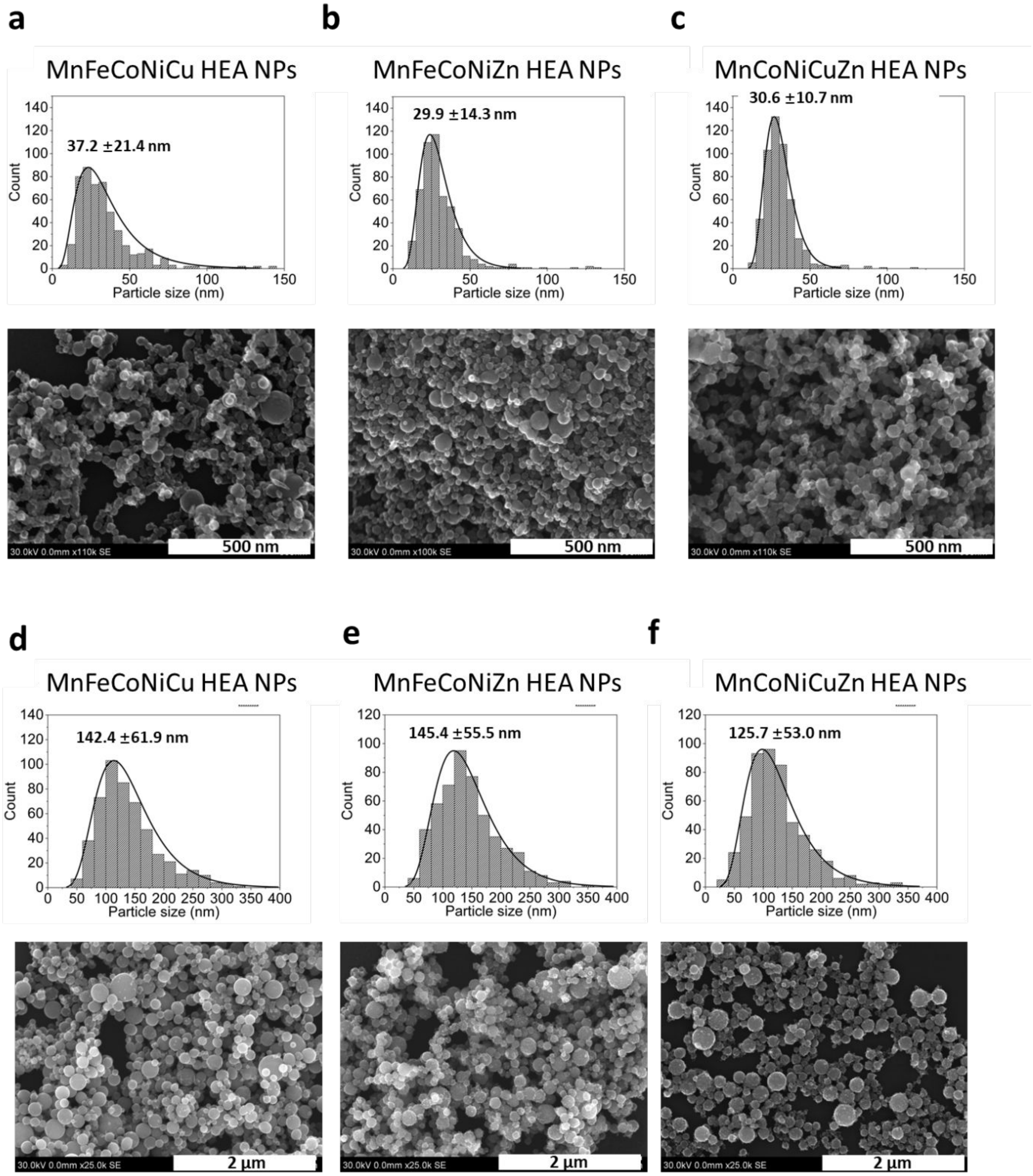

Figure S6. Particle size distributions of the as-produced HEA NPs with the nozzle with SEM images: (a) MnFeCoNiCu HEA NPs. (b) MnFeCoNiZn HEA NPs. (c) MnCoNiCuZn HEA NPs. Particle size distributions of the as-produced HEA NPs without the nozzle with SEM images: (d) MnFeCoNiCu HEA NPs. (e) MnFeCoNiZn HEA NPs. (f) MnCoNiCuZn HEA NPs.

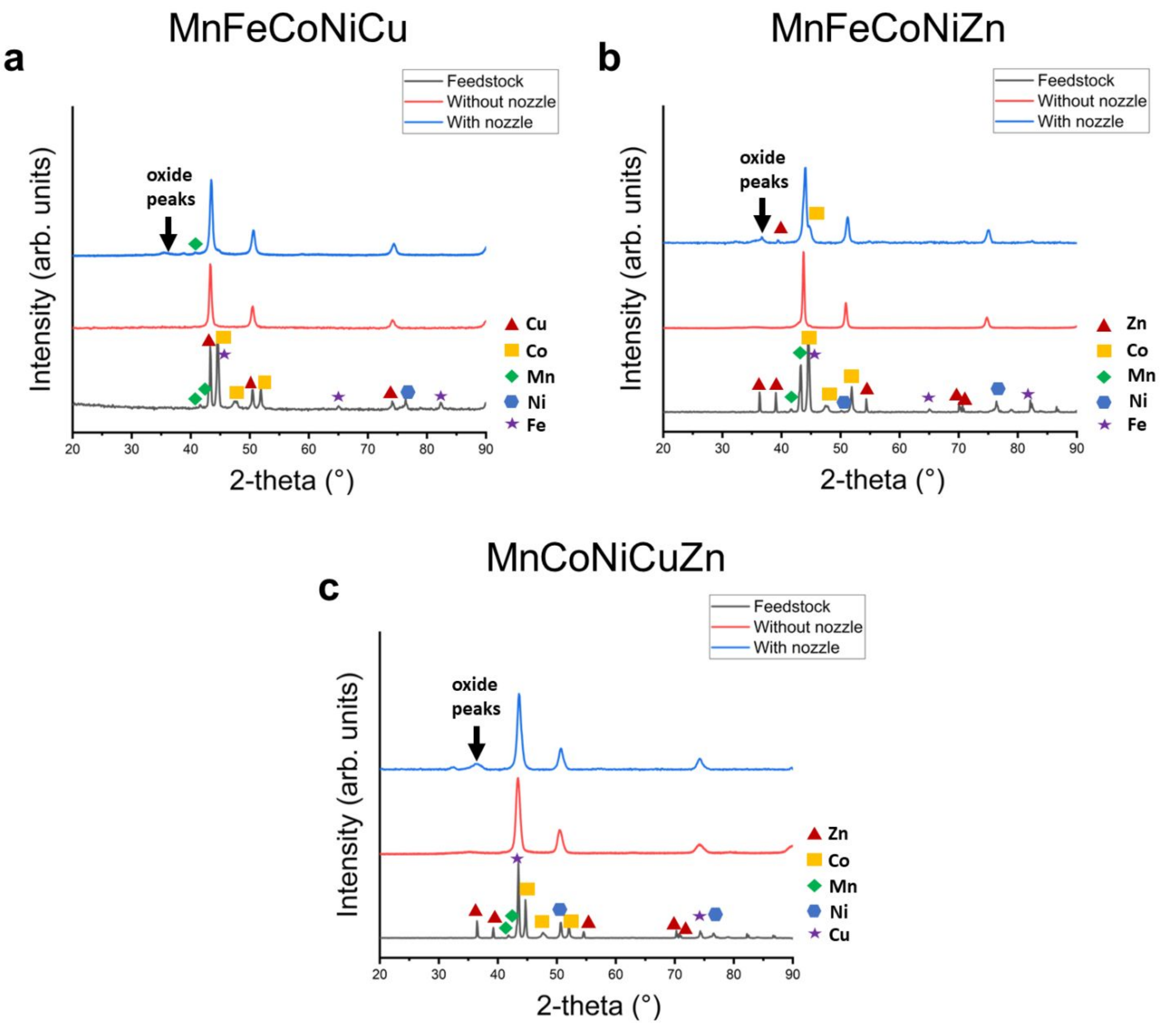

Figure S7. XRD patterns of the feedstock mixtures and the as-produced HEA NPs. (a) MnFeCoNiCu HEA NPs. (b) MnFeCoNiZn HEA NPs. (c) MnCoNiCuZn HEA NPs.

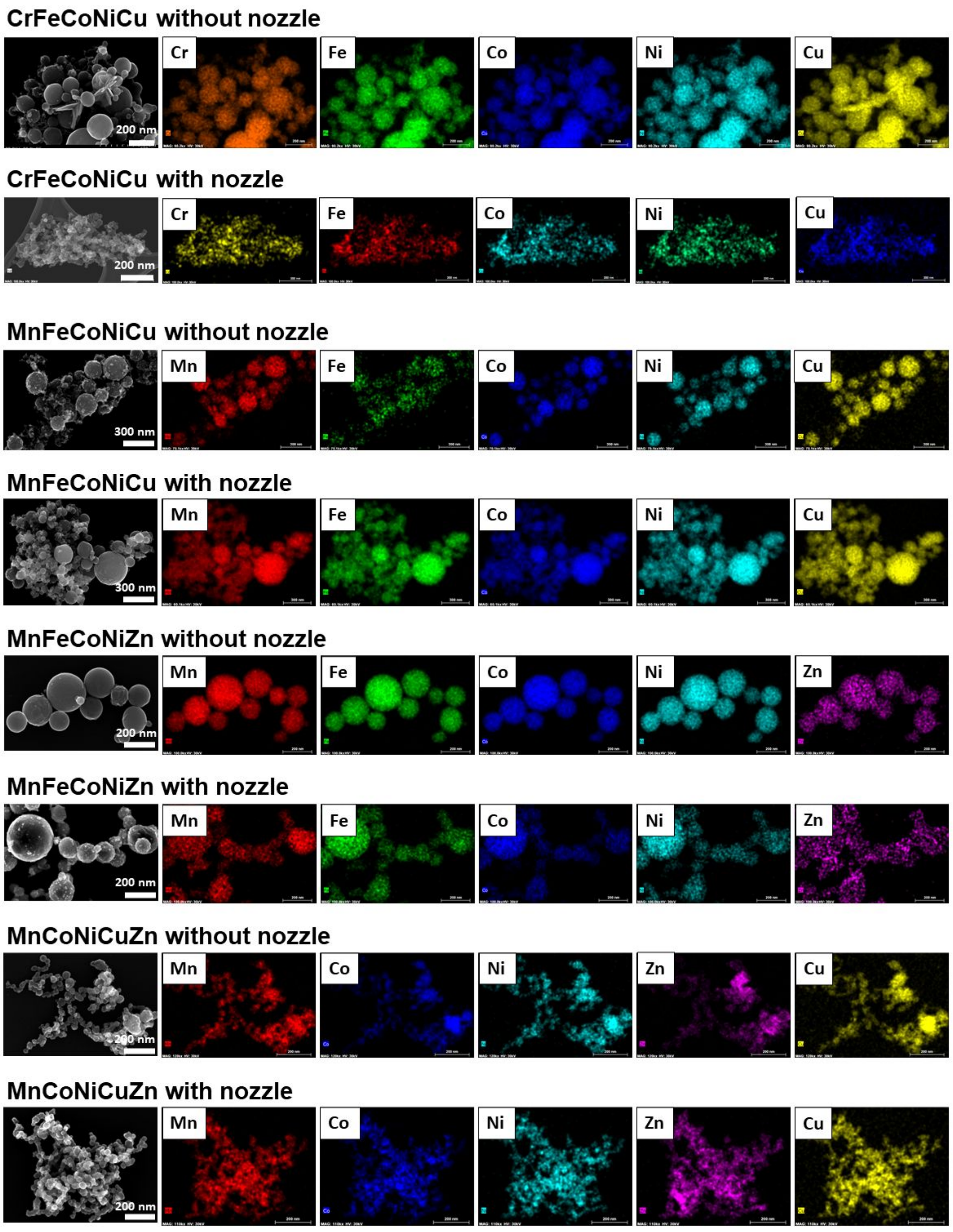

Figure S8. SEM-EDX elemental maps of the HEA NP samples produced with and without the nozzle.

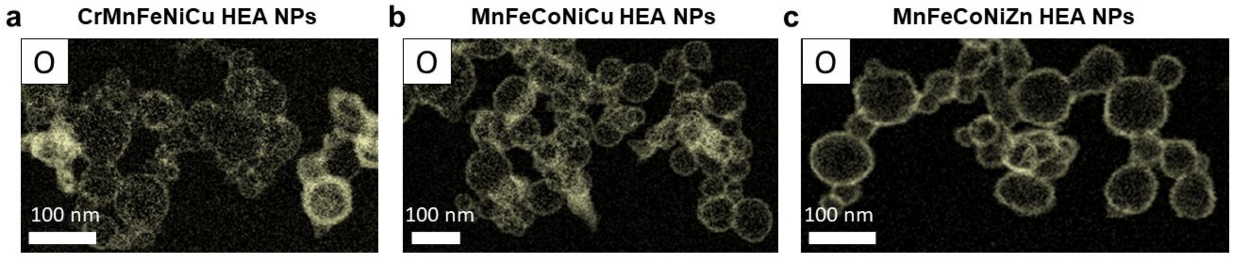

Figure S9. STEM-EELS oxygen maps. (a) CrFeCoNiCu HEA NPs. (b) MnFeCoNiCu HEA NPs. (c) MnFeCoNiZn HEA NPs.

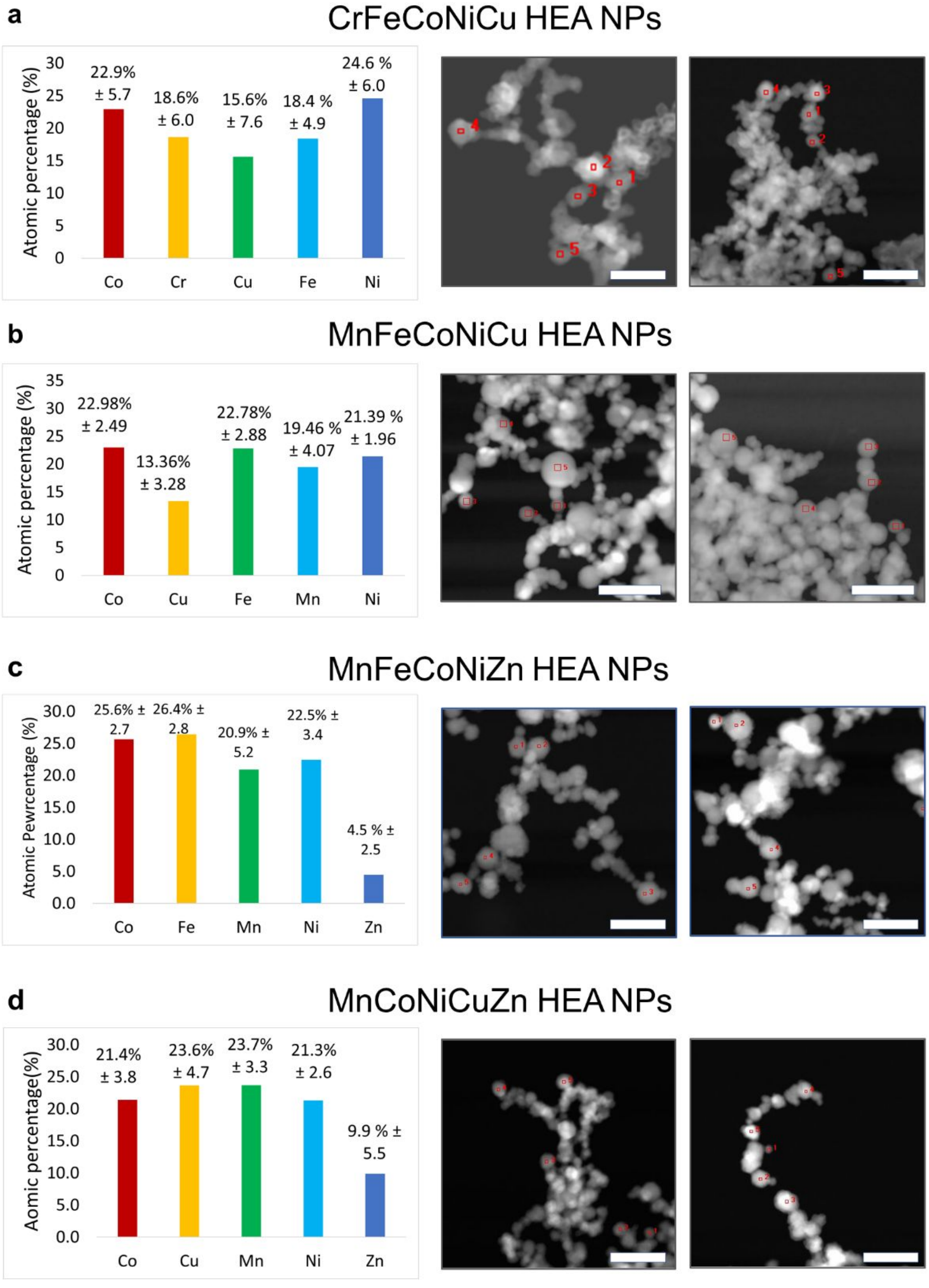

Figure S10. TEM-EDX analysis. Statistical quantification analysis on composition and corresponding HAADF-STEM images of (a) CrFeCoNiCu, (b) MnFeCoNiCu, (c) MnFeCoNiZn, and (d) MnCoNiCuZn HEA NPs. Scale bar, 100 nm.

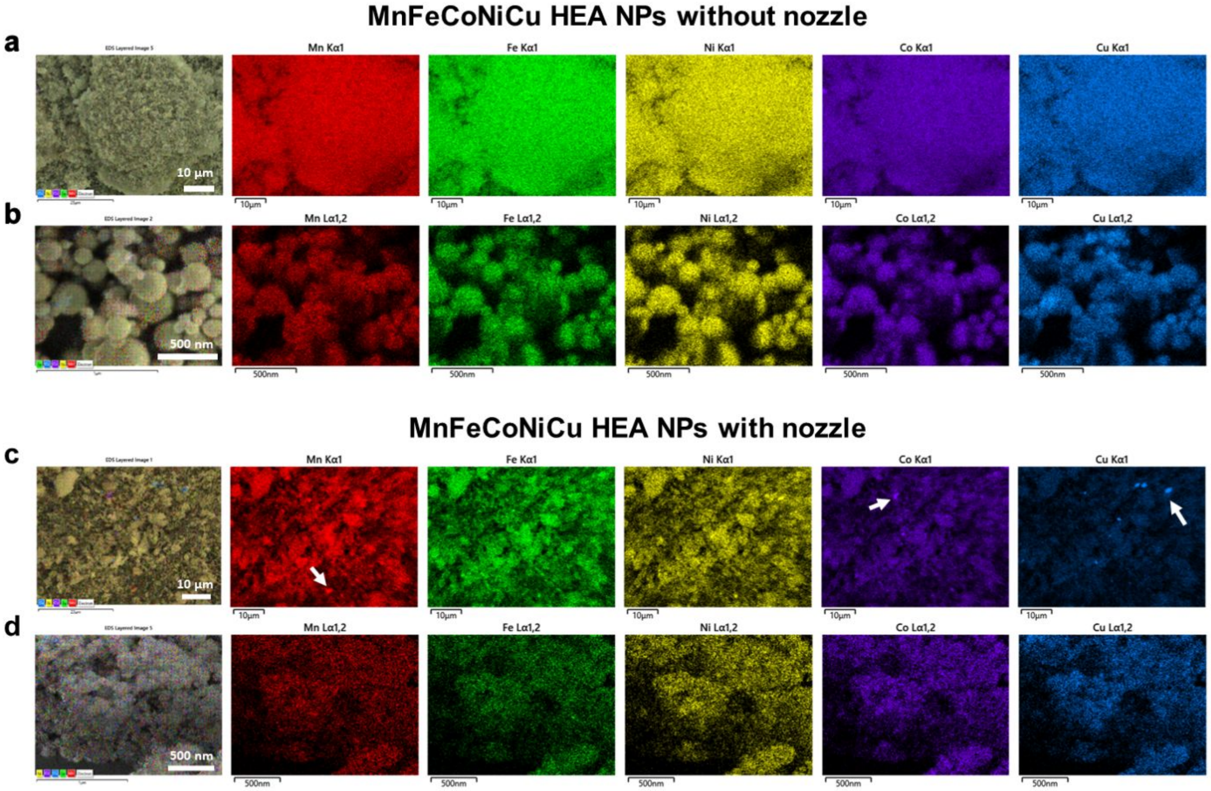

Figure S11. SEM-EDX elemental maps of MnFeCoNiCu HEA NPs. (a) Low magnification (synthesized without nozzle). (b) High magnification (synthesized without nozzle). (c) Low magnification (synthesized with nozzle). (d) High magnification (synthesized with nozzle). Arrows in (c) indicate incompletely vaporized feedstock.

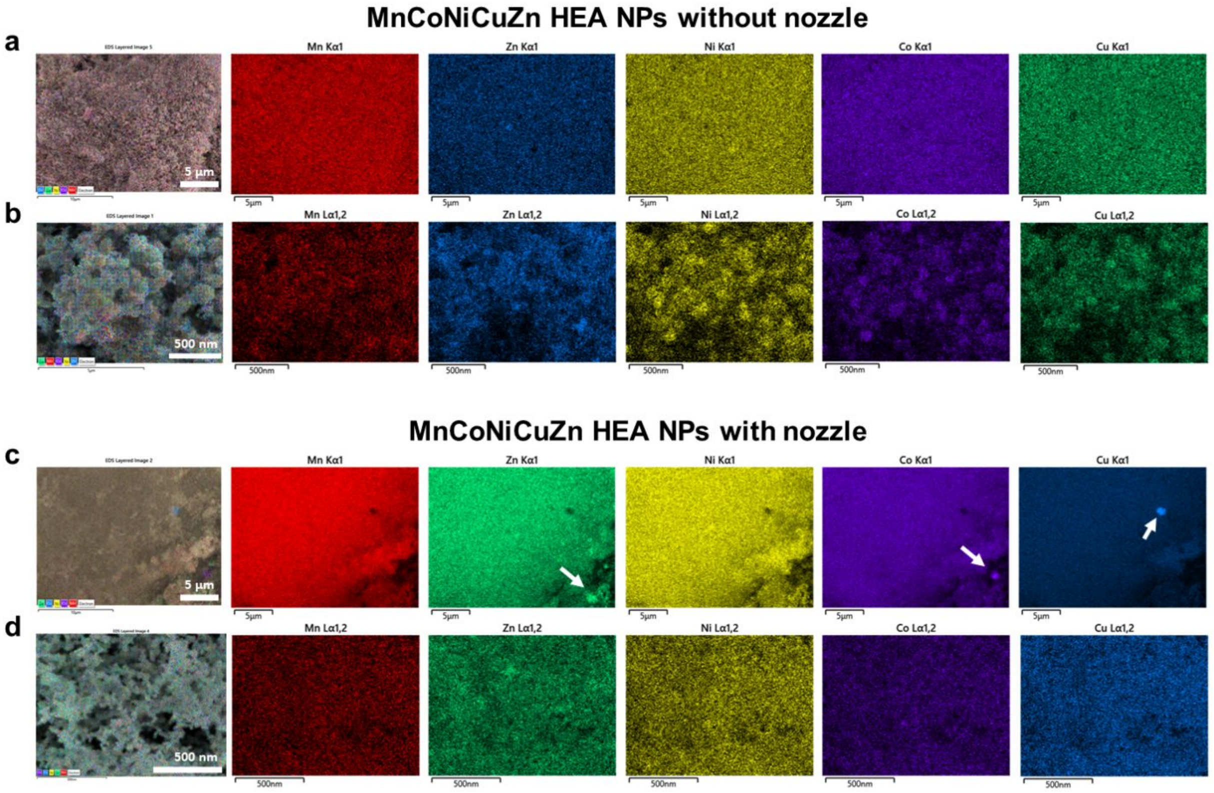

Figure S12. SEM-EDX elemental maps of MnCoNiCuZn HEA NPs. (a) Low magnification (synthesized without nozzle). (b) High magnification (synthesized without nozzle). (c) Low magnification (synthesized with nozzle). (d) High magnification (synthesized with nozzle). Arrows in (c) indicate incompletely vaporized feedstock.

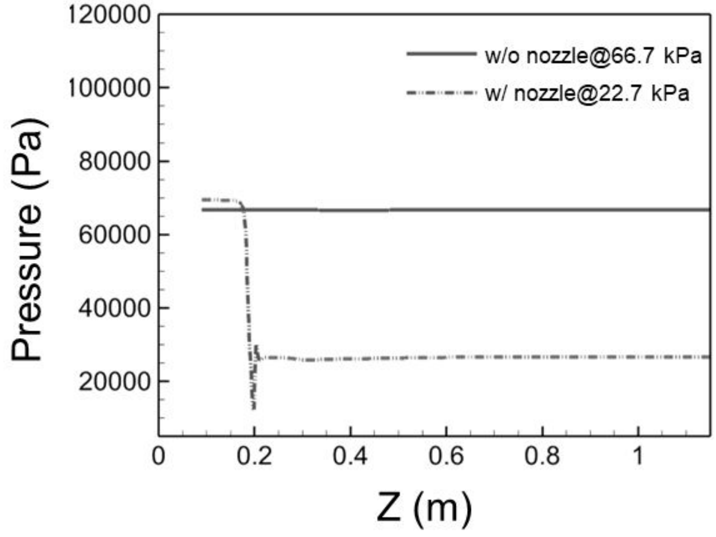

Figure S13. Axial static pressure profiles calculated for different plasma processing conditions.

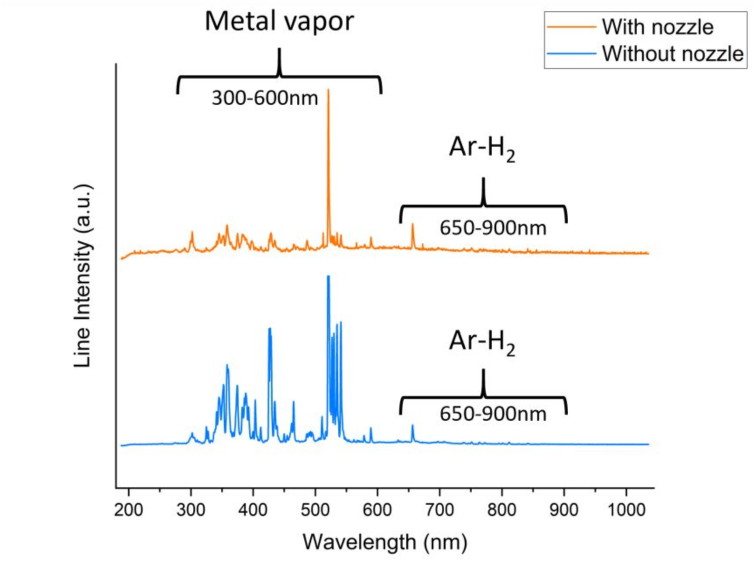

Figure S14. Optical emission spectra measured at Z = 0.49 m from the top of the plasma torch during the synthesis of CrFeCoNiCu HEA NPs.

Table S1. The mixing enthalpies of binary elementary combinations.[1]

	Co	Cr	Cu	Fe	Mn	Ni	Zn
Co	-	-4	6	-1	-5	0	-5
Cr	-4	-	12	-1	2	-7	5
Cu	6	12	-	13	4	4	1
Fe	-1	-1	13	-	0	-2	4
Mn	-5	2	4	0	-	-8	-6
Ni	0	-7	4	-2	-8	-	-9
Zn	-5	5	1	4	-6	-9	-

unit: kJ mol^{-1}

Table S2. Lattice constants of the HEA NPs synthesized with the nozzle.

Sample	Lattice constant (Å)	Crystallite size (nm)	Particle size (nm)
CrFeCoNiCu	3.578	11.324	31.1
MnFeCoNiCu	3.605	13.208	37.2
MnFeCoNiZn	3.565	14.171	29.9
MnCoNiCuZn	3.599	9.571	30.6

Table S3. Lattice constants of the HEA NPs synthesized without the nozzle.

Sample	Lattice constant (Å)	Crystallite size (nm)	Particle size (nm)
CrFeCoNiCu	3.584	17.385	128.5
MnFeCoNiCu	3.613	15.259	142.4
MnFeCoNiZn	3.585	19.911	145.4
MnCoNiCuZn	3.608	8.799	125.7

Table S4. Interplanar spacings and the corresponding lattice constants of the HEA NPs produced.

CrFeCoNiCu (without nozzle)			
hkl	$h^2 + k^2 + l^2$	*d* spacing (Å)	Lattice constant (Å)
111	1.732	2.0701	3.5856
200	2	1.7924	3.5849
220	2.828	1.2666	3.5826
			Average: 3.5843

CrFeCoNiCu (with nozzle)			
hkl	$h^2 + k^2 + l^2$	*d* spacing (Å)	Lattice constant (Å)
111	1.732	2.0655	3.5776
200	2	1.7895	3.5791
220	2.828	1.2648	3.5775
			Average: 3.5781

MnFeCoNiCu (without nozzle)			
hkl	$h^2 + k^2 + l^2$	*d* spacing (Å)	Lattice constant (Å)
111	1.732	2.0881	3.6167
200	2	1.8055	3.6111
220	2.828	1.2767	3.6111
			Average: 3.6130

MnFeCoNiCu (with nozzle)			
hkl	$h^2 + k^2 + l^2$	*d* spacing (Å)	Lattice constant (Å)
111	1.732	2.0812	3.6048
200	2	1.8028	3.6056
220	2.828	1.2745	3.6048
			Average: 3.6050

MnFeCoNiZn (without nozzle)			
hkl	$h^2 + k^2 + l^2$	*d* spacing (Å)	Lattice constant (Å)
111	1.732	2.0680	3.5818
200	2	1.7922	3.5845
220	2.828	1.2686	3.5881
			Average: 3.5848

MnFeCoNiZn (with nozzle)			
hkl	$h^2 + k^2 + l^2$	*d* spacing (Å)	Lattice constant (Å)
111	1.732	2.0527	3.5554
200	2	1.7828	3.5655
220	2.828	1.2638	3.5745
			Average: 3.5652

MnCoNiCuZn (without nozzle)			
hkl	$h^2 + k^2 + l^2$	*d* spacing (Å)	Lattice constant (Å)
111	1.732	2.0819	3.6060
200	2	1.8041	3.6082
220	2.828	1.2764	3.6101
			Average: 3.6081

MnCoNiCuZn (with nozzle)			
hkl	$h^2 + k^2 + l^2$	*d* spacing (Å)	Lattice constant (Å)
111	1.732	2.0727	3.5900
200	2	1.7986	3.5972
220	2.828	1.2763	3.6098
			Average: 3.5990

Table S5. Comparison of theoretical and experimental lattice constants of the HEA NPs synthesized with the nozzle.

	MnFeCoNiCu	CrFeCoNiCu	MnFeCoNiZn	MnCoNiCuZn
Vegard's law	3.538	3.553	3.554	3.552
Cal. (NM)	3.496	3.517	3.487	3.552
Cal. (FM)	3.563	3.563	3.548	3.594
Exp. (XRD)	3.605	3.578	3.565	3.599

Table S6. Mixing enthalpies and mixing entropies of the HEA NPs synthesized with the nozzle.[1,2]

Sample	Atomic ratio	ΔH_{mix} (kJ/mol)	ΔS_{mix} (J/mol)	T_m (K)	Ω
CrFeCoNiCu	18.6:18.4:22.8:24.6:15.6	1.73	13.31	1,651	13.7
MnFeCoNiCu	19.5:22.8:23:21.4:13.4	0.37	13.25	1,667	59.7
MnFeCoNiZn	20.9:26.4:25.6:22.5:4.5	-3.95	12.49	1,668	5.3
MnCoNiCuZn	23.7:21.4:21.3:23.6:9.9	-1.37	13.06	1,495	14.3

$$\Delta H_{mix} = \sum_{i=1,\ i\neq j}^{n} \lambda_{ij} c_i c_j \qquad (1)$$

where $\lambda_{ij} = 4\Delta_{mix}^{AB}$, with Δ_{mix}^{AB} the mixing enthalpy for binary AB alloys. c_i is the atomic percentage of the ith element.

$$\Delta S_{mix} = -R\sum_{i=1}^{n} c_i \ln c_i \qquad (2)$$

where R is the gas constant.

$$T_m = \sum c_i (T_m)_i \qquad (3)$$

$(T_m)_i$ is the melting point of the ith component of the alloy.

$$\Omega = \frac{T_m \Delta S_{mix}}{|H_{mix}|}$$

Table S7. Key features of different HEA NP synthesis methods.

	Particle size	Composition	Yield	Advantage[9]	Limitation[9]
Carbothermal-shock[3]	3-25 nm	Pt, Pd, Co, Ni, Fe, Au, Cu, Sn, Co, Mo, Fe, Ni	10-100 mg (batch)	Fast process; good element inclusiveness and homogeneity	Limited to conductive support; batch process
Aerosol[4]	~100 nm	Pd, Ru, Mo, Sn, Cu, Fe, Co, Ni, Pt	~ few g/h (continuous)	Continuous process; potential for scalable production	Limited to metal salts feedstock dissolve in solvent
Wet-chemical[5]	~3 nm	Au, Pt, Pd, Ru, Rh	~few mg (batch)	Low temperature process; isolated NPs	Potential particle aggregation; batch mode
Microwave-assisted[6]	~12 nm	Pt, Pd, Fe, Co, Ni	~few mg (batch)	High thermal efficiency; isolated NPs with good size and composition control	Difficult to scale up due to limited microwave sources; requires specific support
Solvothermal continuous-flow reactor[7]	2-6 nm	Co, Ni, Cu, Ru, Rh, Pd, In, Sn, Ir, Pt	~0.5 g/h (continuous)	Continuous process, isolated NPs; potential for high-throughput synthesis	Limited to metal salts feedstock in solution
Plasma arc discharge[8]	~100 nm	Co, Cr, Cu, Fe, Ni, Al	1-30 g (batch)	Simple and high production rate	Limited control in composition and particle size
Thermal plasma (this work)	30-130 nm	Co, Cr, Fe, Ni, Cu, Mn, Zn, Mo	5-30 g/h (continuous)	Fast and continuous process; tunable size and microstructure; diverse feedstock; high production rate	Relatively large particle size; high energy consumption; requires specialized equipment

Supplementary References

(1) Takeuchi A.; Inoue A. Calculations of mixing enthalpy and mismatch entropy for ternary amorphous alloys. *Materials Transactions, JIM* **2000**, *41* (11), 1372-1378.

(2) Yang X.; Zhang Y. Prediction of high-entropy stabilized solid-solution in multi-component alloys. *Materials Chemistry and Physics* **2012,** *132* (2-3), 233-238.

(3) Yao, Y.; Huang, Z.; Xie, P.; Lacey, S. D.; Jacob, R. J.; Xie, H.; Chen, F.; Nie, A.; Pu, T.; Rehwoldt, M.; Yu, D.; Zachariah, M. R.; Wang, C.; Shahbazian-Yassar, R.; Li, J.; Hu, L. Carbothermal Shock Synthesis of High-Entropy-Alloy Nanoparticles. *Science* **2018**, 359, 1489–1494.

(4) Yang, Y.; Song, B.; Ke, X.; Xu, F.; Bozhilov, K. N.; Hu, L.; Shahbazian-Yassar, R.; Zachariah, M. R. Aerosol Synthesis of High Entropy Alloy Nanoparticles. *Langmuir* **2020**, 36, 1985–1992.

(5) Liu, M.; Zhang, Z.; Okejiri, F.; Yang, S.; Zhou, S.; Dai, S. Entropy-Maximized Synthesis of Multimetallic Nanoparticle Catalysts via an Ultrasonication-Assisted Wet Chemistry Method under Ambient Conditions. *Advanced Materials Interfaces* **2019**, 6, 1900015.

(6) Qiao, H.; Saray, M. T.; Wang, X.; Xu, S.; Chen, G.; Huang, Z.; Chen, C.; Zhong, G.; Dong, Q.; Hong, M.; Xie, H.; Shahbazian-Yassar, R.; Hu, L. Scalable Synthesis of High Entropy Alloy Nanoparticles by Microwave Heating. *ACS Nano* **2021**, 15, 14928–14937.

(7) Mukoyoshi, M.; Kusada, K.; Zhou, X.; Toriyama, T.; Yamamoto, T.; Murakami, Y.; Kitagawa, H. High-throughput Synthesis of Multi-element Alloy Nanoparticles Using Solvothermal Continuous-flow Reactor. *Faraday Discussion* **2026**, 264, 83-94.

(8) Mao, A.; Xiang, H.; Ran, X.; Li, Y.; Jin, X.; Yu, H.; Gu, X. Plasma Arc Discharge Synthesis of Multicomponent Co–Cr–Cu–Fe–Ni Nanoparticles. *Journal of Alloys and Compounds* **2019**, 775, 1177–1183.

(9) Kar, N.; Skrabalak, S. E. Synthetic Methods for High-Entropy Nanomaterials. *Nature Reviews Materials* **2025**, 10, 638–653.
